# Transcriptional regulation of neuropeptide receptors underlies context‐dependent adaptation in *Drosophila melanogaster*

**DOI:** 10.1002/2211-5463.70107

**Published:** 2025-08-21

**Authors:** SeungHeui Ryu, Yanan Wei, Zekun Wu, Tianmu Zhang, DoHoon Lee, Hadi Najafi, Woo Jae Kim

**Affiliations:** ^1^ Department of Information Convergence Engineering Pusan National University Busan Korea; ^2^ The HIT Center for Life Sciences Harbin Institute of Technology Harbin China; ^3^ Department of Molecular, Cell and Cancer Biology University of Massachusetts Chan Medical School Worcester MA USA; ^4^ Medical and Health Research Institute, Zhengzhou Research Institute of HIT Zhengzhou China

**Keywords:** *Drosophila melanogaster*, neuropeptide, neuropeptide receptor, peptidergic signaling, transcription factor, transcriptional regulation

## Abstract

Neuropeptides (NPs) and their receptors (NPRs) play critical roles in modulating physiological processes and behaviors across species. While the transcriptional regulation of NP genes has been extensively studied, how NPRs contribute to context‐dependent behavioral plasticity remains poorly understood. Here, we investigate the genomic features and expression patterns of NPRs in *Drosophila melanogaster*, leveraging comparative genomics, single‐cell RNA sequencing (scRNA‐seq), transcription factor (TF) network analysis, and empirical validation to uncover the regulatory mechanisms that involve NPRs and play roles in context‐dependent adaptation. We demonstrate that NPR genes exhibit more complex *cis*‐regulatory landscapes, with greater numbers of enhancers compared to NP genes. Also, NPRs are regulated via a broader network of TFs, particularly in response to environmental and physiological cues such as temperature shifts. Through analysis of scRNA‐seq data and qRT‐PCR, we show that the expression level of NPRs is dynamically modulated in a context‐dependent manner, while NP levels remain relatively stable. This “NPR‐biased” gene regulation is evident across diverse combinations of NP‐NPR pairs, with a distinct pattern of TF control in the head and body of *D. melanogaster*. Furthermore, the expression level of NPR genes increases during aging of the fly, suggesting a key role in aging and developmental processes. Our findings highlight the importance of NPR transcriptional control in shaping neuropeptidergic signaling and adaptive behaviors.

## Graphical abstract

Under environmental changes, the expression level of neuropeptide (NP) and neuropeptide receptor (NPR) genes changes to confer context‐dependent adaptation to the model organism *Drosophila melanogaster*. Through finding more regulatory elements in the NPR genes in comparison with their ligands (NPs), we found that NPR‐biased transcriptional regulation is the underlying mechanism in the context‐dependent adaptation process mediated by the NP/NPR signaling pathway.

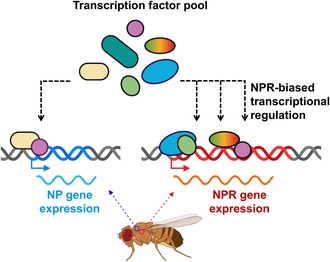

**Abbreviations**
AFCA: aging fly cell atlas
ARRIVE: animal research: reporting of *in vivo* experiments
AS: alternative splicing
CRM: *cis* regulatory module
*D. melanogaster*
: *Drosophila melanogaster*
FCA: fly cell atlas
GPCRs: G‐protein coupled receptors
GRN: gene regulatory network
kb: kilobase pairs
NP: neuropeptide
NPR: neuropeptide receptor
qRT‐PCR: quantitative reverse transcription PCR
RPKM: Reads Per Kilobase per Million mapped reads
scRNA‐seq: single‐cell RNA‐sequencing
SEM: standard error of the mean
TCC: total cell count
TF: transcription factor
TFBS‐HSA: TF binding sites HOT spot analysis
TPM: transcripts per million mapped reads
UMI: unique molecular identifier

The ability of an organism to adapt to a changing environment relies remarkably on dynamic changes in the expression and/or activity of the genes that sense, process, and respond properly to the external and internal cues [1, 2, 3]. With the capability of signal perception from the environment and also releasing modulatory biomolecules such as neuropeptides (NPs), the nervous system plays a fundamental role in context‐dependent adaptation [4].

As their name implies, NPs are synthesized and released by neurons and bind to their receptors (neuropeptide receptors or NPRs) through which they modulate the physiology and function of both neural and non‐neural tissues [5]. NP expression is crucial in regulating physiological processes and behaviors in many organisms, including humans [6]. These molecules affect numerous functions such as feeding, reproduction, metabolism, and pain perception. A cooperative relationship between NPs and their receptors (NPRs) forms a cascade of signaling networks, essential for maintaining homeostasis and adaptation against environmental changes [7, 8, 9, 10, 11]. Given the widespread expression pattern of NPRs across tissues, NPs can control essentially all aspects of physiology and behavior in an organism.

The multifunctional nature of NPs potentially arises from several factors [12]: First, “receptor specificity and heterogeneity” meaning that each NP can bind to different receptor subtypes or complexes on various target cells, resulting in distinct responses. Therefore, the variety of NPRs and their differential expression pattern across neural circuits and tissues contributes to this multifunctionality. Second, “plurichemical transmission” in which multiple chemical messengers, including NPs, can be released, each having different dynamics and/or affinity to their receptors, leading to varied outcomes. Therefore, the synergic and/or antagonistic relationship between the released NPs and other neurotransmitters or hormones in the synaptic cleft can modulate postsynaptic responses differently [13, 14]. Third, “cellular context and local environment” indicating that local conditions, such as enzymes and transporters, can modulate NP effects. In fact, the environment where a NP is released can influence its bioavailability, degradation, or affinity to its receptor(s), hence resulting in different outcomes [15, 16]. Fourth, “developmental and experience‐dependent plasticity,” which suggests that timing and experience‐related changes in NP sensitivity and expression can lead to different responses based on environmental context.

Theoretically, NP and NPR proteins exert their biological functions in pairs. However, peptide–receptor interactions are often much more complex than a simple one peptide‐one receptor paradigm [5]. Due to varied expression levels and distribution patterns of NPR genes across different tissues and/or cell types, their expression might be under the control of tight and programmed regulatory mechanisms that remain to be investigated. Elucidation of the regulatory factors specific to NPs and NPRs will expand our knowledge in the understanding of the molecular basis of the phenotypes caused by NP and NPR functions and/or their relevant signaling pathways.

Studies have primarily examined the transcriptional regulation of NP genes to understand their roles in the nervous system. While insights into the functionality and expression pattern of NP genes have been gained, the approaches undertaken by previous studies have limitations in explaining the diverse NP functions.

*Drosophila melanogaster* (*D. melanogaster*) offers a unique and valuable model for studying the transcriptional regulation of NPs and NPRs [8, 9, 10, 11, 17]. Like nucleic acid and amino acid sequence conservation of NPs and their receptors (NPRs), their related molecular networks and mechanisms are also evolutionarily conserved across a wide variety of species; hence, they are going to be considered as promising therapeutic targets (e.g., drug targets) for human diseases [18]. For instance, analysis of the *D. melanogaster* genome resulted in the identification of 44 NPR genes, with many shown to have orthologues in vertebrates. Also, neuropeptide ligands of these receptors have been identified in *Drosophila* and other insects [19, 20]. A well‐characterized NP‐NPR pair in humans with evolutionarily conserved sequences and functions is the family of neuropeptide Y genes (NPY, PYY, and PP) with their cognate receptors (hY_1_R, hY_2_R, hY_4_R, and hY_5_R). They regulate physiological and behavioral characteristics such as food intake and anxiety, as well as human diseases such as obesity, mood disorders, and cancer [21]. Also, the homeostatic maintenance of blood sugar levels is mediated by insulin‐like peptides and glucagon‐like peptide in *Drosophila*. Such evolutionary conservation in both structure and function has also been shown for *Drosophila* neuropeptide F (NPF) and mammalian neuropeptide Y (NPY) [22]. Nevertheless, while numerous NP and NPR genes were discovered and functionally annotated in a wide variety of organisms, the detailed mechanisms underlying their spatiotemporal expression control are not well‐understood.

In this study, we utilized an unprecedented approach combining both computational and empirical methods to study the features of NP regulatory networks in pair with their receptors. The multifunctionality of NPs in modulating various physiological and behavioral responses is complex when viewed solely through peptide expression, since a single NP can stimulate different effects in distinct neural circuits and/or environments. To address this, we propose a hypothesis that emphasizes the transcriptional control of NPRs rather than the NPs themselves [8, 20, 23, 24, 25, 26, 27]. We suggest that controlling the transcription of NPR genes is crucial for context‐dependent neuropeptidergic modulation of physiology and behavior.

Here, we demonstrate that the transcriptional regulation of NPRs may evolve to influence complex behavioral plasticity, utilizing the most recent releases of single‐cell RNA sequencing (scRNA‐seq) data in FlySCope [28], and further supporting evidence obtained by regulatory network analyses and empirical validation.

## Materials and methods

### *Drosophila* data collection and analysis

This study utilized publicly available databases and resources to systematically collect data on transcription factor binding sites (TFBS), enhancers, and biological information related to neuropeptides (NPs) and neuropeptide receptors (NPRs) in *D. melanogaster*. Using the list of NPs and NPRs from Table 1, which is based on the most recent research on fly NPs [29], we searched in the FlyBase [30, 31] for each gene and manually examined presence of the transcription factor binding sites (TFBS) identified through hot spot analyses (TFBS‐HSA) [32]. This was done using the JBrowse tool available on FlyBase, which allows providing detailed visualization of genetic data [30, 31, 33, 34]. These genomic regions were obtained through ChIP‐Chip and ChIP‐Seq analysis of 41 TFs in *D. melanogaster* embryo; however, there is a great concordance between TFBS‐HOT regions and open chromatin state and increased nucleosome turnover, both signs of active gene expression regulation [35]. However, despite their high occupancy of TFs, some TFBS‐HOT spots were found to be depleted from *cis* elements such as enhancers, suggesting additional mechanisms for these elements other than direct transcriptional control of the genes [35, 36]. Therefore, in addition to TFBS‐HOT spots in our study, other classes of regulatory elements such as *cis*‐regulatory modules (CRMs) were investigated for both NP and NPR genes. These “transcriptional regulatory elements” were collected from the REDfly database [37, 38, 39], which provides curated information on the *Drosophila* regulatory elements. Despite TFBS‐HOT regions, CRMs were empirically tested and confirmed as functional *cis* regulatory elements, which are closely related to transcriptional control of their surrounding genes [40]. Like TFBS‐HOT spots, two features of CRMs including “number” and “genomic length” were investigated in this study for both NP and NPR genes.

**Table 1:** List of neuropeptides (NPs) and neuropeptide receptors (NPRs) in *Drosophila melanogaster*.

Raw #	NPs		NPRs	
	Official symbol	FlyBase ID	Official symbol	FlyBase ID
1	Akh	FBgn0004552	AkhR	FBgn0025595
2	AstA	FBgn0015591	AstA‐R1	FBgn0266429
3	AstA	FBgn0015591	AstA‐R2	FBgn0039595
4	AstC	FBgn0032336	AstC‐R1	FBgn0036790
5	AstC	FBgn0032336	AstC‐R2	FBgn0036789
6	burs	FBgn0038901	rk	FBgn0003255
7	Capa	FBgn0039722	CapaR	FBgn0039396
8	Capa	FBgn0039722	PK1‐R	FBgn0038201
9	CCHa1	FBgn0038199	CCHa1‐R	FBgn0050106
10	CCHa2	FBgn0038147	CCHa2‐R	FBgn0033058
11	CNMa	FBgn0035282	CNMaR	FBgn0053696
12	CRZ	FBgn0013767	CrzR	FBgn0036278
13	CCAP	FBgn0039007	CCAP‐R	FBgn0039396
14	Dh31	FBgn0032048	Dh31‐R	FBgn0052843
15	Dh31	FBgn0032048	hector	FBgn0030437
16	Dh44	FBgn0012344	Dh44‐R1	FBgn0033932
17	Dh44	FBgn0012344	Dh44‐R2	FBgn0033744
18	ETH	FBgn0028738	ETHR	FBgn0038874
19	FMRFamide	FBgn0000715	FMRFaR	FBgn0035385
20	Gpb5	FBgn0063368	Lgr1	FBgn0016650
21	Hug	FBgn0028374	PK2‐R2	FBgn0038139
22	Hug	FBgn0028374	PK2‐R1	FBgn0038140
23	Ilp1	FBgn0044051	InR	FBgn0283499
24	ilp2	FBgn0036046	InR	FBgn0283499
25	ilp3	FBgn0044050	InR	FBgn0283499
26	ilp4	FBgn0044049	InR	FBgn0283499
27	ilp5	FBgn0044048	InR	FBgn0283499
28	ilp6	FBgn0044047	InR	FBgn0283499
29	ilp7	FBgn0044046	Lgr4	FBgn0085440
30	ilp8	FBgn0036690	Lgr3	FBgn0039354
31	Lk	FBgn0028418	Lkr	FBgn0035610
32	Lst	FBgn0034140	PK1‐R	FBgn0038201
33	Ms	FBgn0011581	MsR1	FBgn0035331
34	Ms	FBgn0011581	MsR2	FBgn0264002
35	natalisin	FBgn0085417	TkR86C	FBgn0004841
36	NPF	FBgn0027109	NPFR	FBgn0037408
37	Nplp1	FBgn0035092	Gyc76C	FBgn0266136
38	PDF	FBgn0023178	Pdfr	FBgn0260753
39	Proctolin	FBgn0045038	Proc‐R	FBgn0029723
40	PTTH	FBgn0013323	torso	FBgn0021796
41	RYamide	FBgn0085512	Rya‐R	FBgn0004842
42	Acp26Aa	FBgn0002855	SPR	FBgn0029768
43	sNPF	FBgn0032840	sNPF‐R	FBgn0036934
44	SIFa	FBgn0053527	SIFaR	FBgn0038880
45	Dsk	FBgn0000500	CCKLR‐17D1	FBgn0259231
46	Dsk	FBgn0000500	CCKLR‐17D3	FBgn0030954
47	Tk	FBgn0037976	TkR99D	FBgn0004622
48	Trissin	FBgn0038343	TrissinR	FBgn0085410

Using “ReMap” collection from the UCSC genome browser (*D. melanogaster* genome, BDGP Release 6 + ISO1 MT/dm6) [41], regulon profiles of five well‐documented NP/NPR pairs were investigated. ReMap illustrates a comprehensive atlas of regulatory elements in the respective genes, which consists of a large‐scale integrative analysis of all public ChIP‐seq data for more than 550 transcriptional regulators from 1205 datasets including GEO, ArrayExpress, and ENCODE, which also covers all stages of *D. melanogaster* development [42].

Single‐nucleus transcriptomic data for adult *D. melanogaster* were sourced from the Fly Cell Atlas (FCA) [28]. This dataset provided crucial insights into the gene expression pattern of NP and NPR genes at single‐cell resolution as well as their expression pattern during different developmental stages of the fly. In this platform, the SCope [43] and ASAP [44] were used to explore the tissue specificity of gene expression. Aging‐related transcriptional regulatory patterns were analyzed using the Aging Fly Cell Atlas (AFCA) dataset [45], which confers insights into the evolution of transcription factor (TF) networks at cellular resolution regulating NP and NPR genes during aging. These datasets collectively facilitated the investigation of age‐dependent molecular mechanisms in *D. melanogaster*. All datasets were curated, filtered, and validated to ensure consistency and relevance to the study's objectives.

### Construction of TF‐NP and TF‐NPR regulatory networks

The regulatory networks of “transcription factor–neuropeptide” (TF‐NP) and “transcription factor–neuropeptide receptor” (TF‐NPR) were constructed using data from the Fly Cell Atlas [28]. A total of 17 loom files containing tissue‐specific single‐nucleus RNA sequencing (snRNA‐seq) data were downloaded and processed using the Python package SCANPY [46]. It includes annotation information of tissues, cells, genes and their expression level, and motifs as well as their expression level across cells. Key parameters of the curated data include: Tissue types (*n* = 17), Genes (*n* = 16 373), Cells (*n* = 507 827), Transcription Factors (TFs) (*n* = 565), and Gene expression records (~120 million). The resulting MySQL database, comprising around 21 GB of data, enabled querying of gene expression patterns across the entire *D. melanogaster* dataset, beyond tissue‐specific constraints, using SQL commands. Missing genetic information was supplemented using RNA‐Seq RPKM values from the “gene_rpkm_matrix_fb_2021_06.tsv” file downloaded from FlyBase (https://flybase.org/). These data, originating from FlyAtlas2 [30], include RNA‐Seq and miRNA‐Seq measurements across a wide range of tissues, offering valuable insights into the pattern of gene expression for the gene(s) of interest.

To construct the TF regulatory networks, we processed data of TFs and their “MotifRegulon,” and “MotifRegulonAuc” information generated by the AUCell algorithm from the Fly Cell Atlas [28]. AUCell uses a multistep process comprising: first, co‐expression gene expression analysis between TFs and NP/NPR genes across single cells; then performs motif analysis of the co‐expressed TFs with genes to filter and visualize only the high‐score TF‐NP or TF‐NPR interactions. Also, the resultant gene regulatory networks (GRNs) by AUCell can be further classified based on their cell types. Therefore, a robust network of TF‐NP and TF‐NPR interactions will be built, together with additional “regulon” information including their associated cell types and motif classes [47]. MotifRegulon provides the information of tissues and genes, which are regulated by TFs. MotifRegulonAuc provides a numeric value for the MotifRegulon of TFs expressed in each cell, useful for tissue‐ and/or cell type‐enrichment analyses. Using this information, we can find the NP and NPR genes expressed in each tissue and/or cell type, along with their associated TFs. Therefore, the networks of TF‐NP and TF‐NPR together with their tissue‐ and cell type‐specificity information were obtained.

Regulatory networks between genes and TFs were analyzed using the following steps: First, 10 NPs and 13 NPRs were selected (Table 2). The cells co‐expressing both NP and NPR genes were excluded to maximize the distinction between their regulatory networks. Cells where TFs actively regulate transcription were identified, and the information of the NP and NPR genes regulated by TFs in specific tissues was extracted. TF regulatory networks were built for the cells expressing NPs and their relevant TFs. Similarly, TF regulatory networks were constructed for cells expressing NPRs and their associated TFs. The resulting network graphs allowed us to clearly visualize the differences in the regulatory modules between the TFs that are differentially linked to NPs and NPRs. These data were implemented in Python, and the python packages *matplotlib* [48] and *NetworkX* (https://github.com/networkx/networkx) were utilized for visualization and analysis.

**Table 2:** List of NP–NPR pairs used for network analysis with TFs.

NPs	NPRs
AstA	AstA‐R1
	AstA‐R2
AstC	AstC‐R1
	AstC‐R2
Capa	CapaR
Crz	CrzR
FMRFa	FMRFaR
NPF	NPFR
Natalisin	PK1‐R
	TkR86C
Pdf	Pdfr
SIFa	SIFaR
sNPF	sNPF‐R

### TF regulatory networks during aging of *Drosophila melanogaster*

Single‐cell RNA sequencing (scRNA‐seq) data from the *D. melanogaster* were obtained from the Aging Fly Cell Atlas website (https://hongjielilab.org/afca/), comprising raw unique molecular identifier (UMI) counts for 566 254 cells extracted from the provided “.h5ad” file. The data can be easily visualized through Shiny, provided by Li et al. [45] (https://hongjielilab.shinyapps.io/AFCA/). To infer TF regulatory networks, we applied SCENIC v1.3 [47], through which the TF regulons and importance of the pairs at each time point were predicted based on co‐expression modules, identified by GENIE3 [49] with default parameters. The resulting data were imported into R for visualization and downstream analyses.

### Other resources

Anatomical illustrations for internal and sensory organs of *D. melanogaster* were sourced from [50, 51], respectively.

### Fly stock and husbandry

*Drosophila melanogaster* Canton‐S line was raised on cornmeal‐yeast medium at similar densities to yield adults with similar body sizes. Flies were kept in 12 h light: 12 h dark (LD) cycles at 25 °C (ZT 0 is the beginning of the light phase, ZT12 beginning of the dark phase) as previously described [52, 53, 54].

### Ethics approval and statement of animal research compliance

All animal experiments reported by this study were conducted in compliance with the ARRIVE guidelines and adhered to the U.K. Animals (Scientific Procedures) Act, 1986 and associated guidelines, EU Directive 2010/63/EU for animal experiments, or the National Research Council's Guide for the Care and Use of Laboratory Animals [55].

### RNA extraction and cDNA synthesis

RNA was extracted from 50 preparations of the Canton‐S fly line per condition (50 males and 50 virgin females) using the RNA isolation kit (Vazyme, Nanjing, China, RC202‐01) for extracting total RNAs separately from the head and body parts of *D. melanogaster* following the manufacturer's protocol. For the temperature shift assay, flies with different ages (1, 3, and 5 days post eclosion) were reared in different temperatures: one day in 25 °C and 29 °C, and 2 h in 37 °C prior to RNA extraction. After these incubations, the head and body segments of the flies were separated by collecting them in liquid nitrogen and vortexing until the heads detached from bodies prior to immediate RNA extraction.

### Quantitative reverse transcription polymerase chain reaction (qRT‐PCR)

qRT‐PCR was performed with SYBR Green qPCR Master Mix kit (Selleckchem) in the RT‐qPCR kit (SparkJade) for a total of 40 cycles (94 °C for 20 s, 60 °C for 20 s, 72 °C for 30 s). The *Glyceraldehyde‐3‐phosphate dehydrogenase* (*GAPDH*) gene was used as an internal reference gene, and the mRNA expression levels of NP and NPR genes were calculated using the 2^−ΔΔCT^ method. Primers for amplifying the genes in qRT‐PCR were: *SIFa*, F: 5′‐ACTGCAAGATGGCTCTTCG‐3′; R: 5′‐CGGCATTTCCACATTCAGTC‐3′; *sNPF*, F: 5′‐GTGTTCCTCAGTTCGAGGCAA‐3′; R: 5′‐AGTTCAAAAGCGAGTTGTACCA‐3′; *Crz*, F: 5′‐GAAACTGTGTCCCCGGTTCG‐3′; R: 5′‐AGAGTTGCTCAGTCTGGGATG‐3′; *SIFaR*, F: 5′‐GGGCACGGTTCTCACTACG‐3′; R: 5′‐CGATAGGTTCAGCAAGTTGGAA‐3′; *sNPF‐R*, F: 5′‐AACTGGTTGTGAATGATCCCG‐3′; R: 5′‐CCAACTGGAGCCTAACGTCG‐3′; *CrzR*, F: 5′‐AATCCGGACAAAAGGCTGGG ‐3′; R: 5′‐AGGTGGAAGGCACCGTAGAT‐3′; *AstA*, F: 5′‐TCCCTTCACGCCCACCTCCT‐3′; R: 5′‐TACCGCTCCACCCGCTTGTC‐3′; *AstA‐R1*, F: 5′‐CGCAGAGTCACGAAAGGG‐3′; R: 5′‐CGCCACCACATGGGATAT‐3′; *AstA‐R2*, F:5′‐CCAACCTGATGATTGTCAATCTGGC‐3′; R: 5′‐GGTAATGTTCTCCGTCCTCATCATC‐3′. qRT‐PCRs were performed in triplicate, and the specificity of each reaction was evaluated by dissociation curve analysis. Each experiment was replicated three times. PCR results were recorded as threshold cycle numbers (Ct). The relative expression levels of each NP and NPR gene were calculated by normalizing against the expression level of *GAPDH* gene in the sample [56]. The results were presented as the mean ± SEM of three independent experiments.

### Statistical tests

When the dataset passed the test for normal distribution (Kolmogorov–Smirnov tests, *P* > 0.05), we used two‐sided Student's *t*‐tests, using the mean ± SEM and *P*‐values for comparisons. Statistically significant differences are represented by asterisks in the graphs (****P < 0.0001, ***P < 0.001, **P < 0.01, *P < 0.05), while the nonsignificant ones are denoted by the “ns” in graphs. All analyses were performed in GraphPad (Prism).

Besides traditional *t*‐test for statistical analyses, we added “estimation statistics” for all analyses in two‐group comparing graphs. In short, estimation statistics is a simple framework that—while avoiding the pitfalls of significance testing—uses familiar statistical concepts: means, mean differences, and error bars. More importantly, it focuses on the effect size of one experiment and/or intervention, as opposed to significance testing [57]. In comparison with typical Null Hypothesis Significance Testing (NHST) plots, estimation graphics have the following five significant advantages (1) avoiding false dichotomy, (2) displaying all observed values, (3) visualizing estimate precision, (4) showing mean difference distribution, and, most importantly, (5) by focusing on an effect size, the difference diagram encourages quantitative reasoning about the system under study [58]. Thus, we conducted a reanalysis of all our two‐group datasets using both standard *t*‐tests and estimation statistics. In 2019, the Society for Neuroscience journal eNeuro instituted a policy recommending the use of estimation graphics as the preferred method for data presentation [59].

For datasets that did not exhibit normal distribution, as determined by the Kolmogorov–Smirnov test, we employed nonparametric statistical methods to analyze the data. Specifically, we utilized the Mann–Whitney U test to compare the distributions between two independent groups [60]. This test is appropriate for ordinal data and does not assume a normal distribution, making it a robust choice for our analysis. The results of these comparisons were visualized using rank plots, which provide a graphical representation of the data distribution and the test statistics [49].

## Results

### Comparative *cis*‐regulatory landscape of NP and NPR genes in *Drosophila melanogaster*

**Fig. 1:**
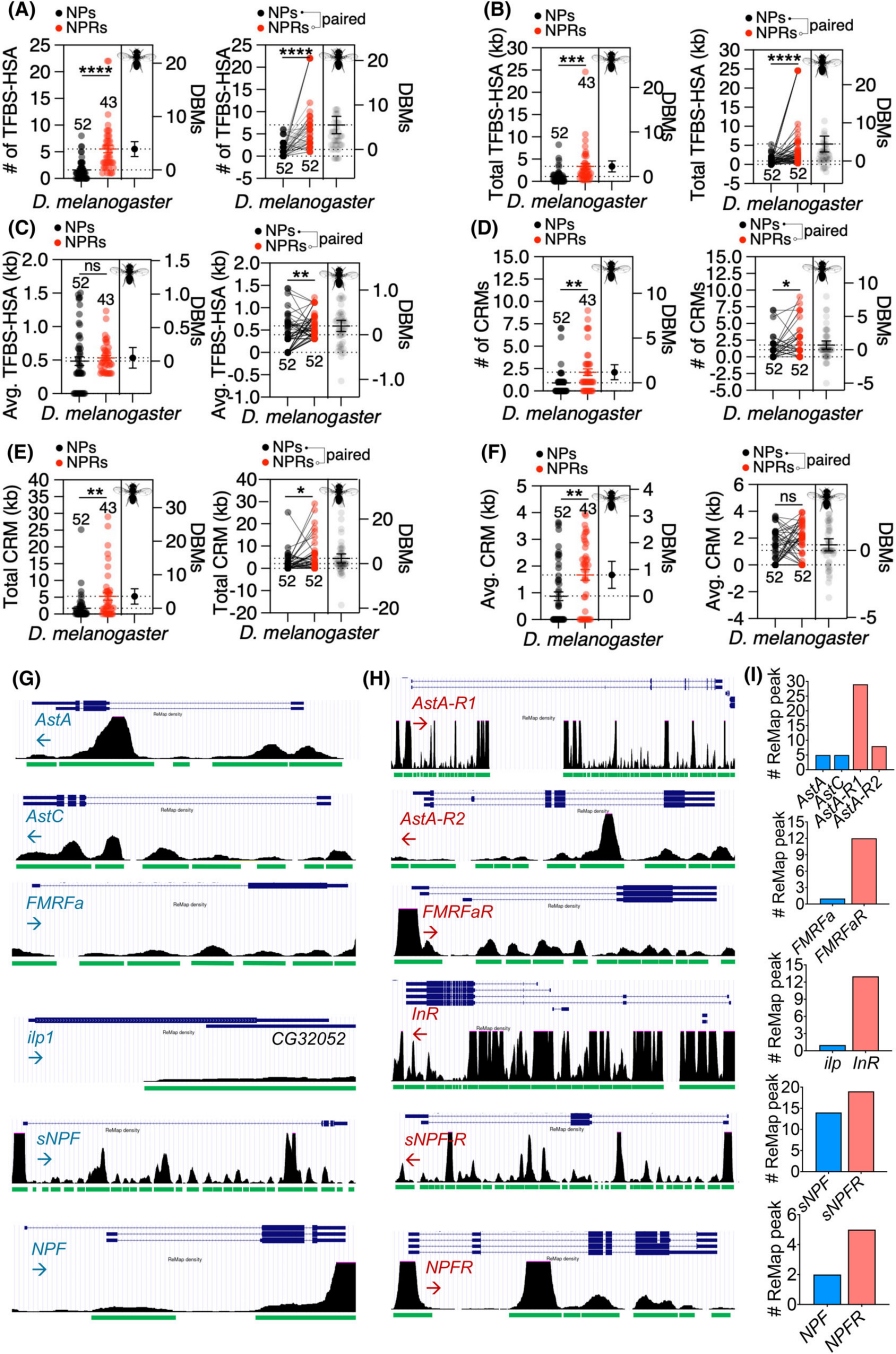
NPR genes exhibit a more complex regulatory architecture than NP genes in *Drosophila melanogaster*. (A, B) Corresponding regulatory landscape in *D. melanogaster*, displaying the number (#) and total length (kilobase pairs or ‘kb’) of transcription factor binding site hotspot areas (TFBS‐HSA) for NP and NPR genes, along with the difference between means (DBMs) between NP and NPR genes. (C) Mean lengths of transcription factor binding site hotspot areas (TFBS‐HSA) in *D. melanogaster* for NP and NPR genes. (D, E) Number and total length of *cis*‐regulatory modules (CRMs) identified in *Drosophila* NP and NPR genes, indicating variation in regulatory complexity between the two gene types. (F) Mean length of *cis*‐regulatory modules (CRMs) in the *D. melanogaster* NP and NPR genes. For all graphs, the mean value and standard error are labeled within the dot plot (black lines). DBMs stands for ‘Difference Between Means’ and used for the evaluation of estimation statistics [57] (See Materials and Methods). Asterisks represent significant differences (*P < 0.05, **P < 0.01, ***P < 0.001, ****P < 0.0001), as revealed by the unpaired Student's *t*‐test, and ‘ns’ represents non‐significant differences. Analyses were performed in both paired and unpaired fashions, as indicated on top of graphs. (G–I) UCSC genome browser snapshots of the indicated *D. melanogaster* NP/NPR genes, showing a greater number of regulatory elements within the NPR genomic regions versus NPs, obtained from the ReMap database. Peaks are adjusted to the same scale (0–100) for all genes, and the frequency (#) of the ReMap peaks for each gene is illustrated in panel “I.”

The abundance (number) and total length (kilobase pair, kb) of transcription factor binding sites (TFBSs) in the genomic regions of NP and NPR genes were investigated through transcription factor hot spot analysis (TFBS‐HSA) in FlyBase, and results showed a markedly higher number and length of these elements within the *cis*‐regulatory regions of NPR genes rather than these elements in NP genes, irrespective of whether they were analyzed in unpaired or paired modes (Table 1; Fig. 1A,B). However, despite discernible differences in the quantity of TFBS‐HSA hits for NP and NPR genes, the mean (average) length of these TFBS‐HSA elements was found to be similar between the two gene classes (Fig. 1C). This observation implies that the diversity of TFBS components, rather than their linear dimensions, may exert a crucial influence on the modulation of gene transcription.

*Cis*‐regulatory modules (CRMs) and noncoding DNA segments are also essential for gene expression regulation [61]. As essential regulatory *cis* elements outside the core promoter, CRMs execute their roles in gene expression regulation through spatiotemporal control of chromatin in *cis* [37, 38, 39]. We extended our analyses to find the “empirically validated” CRMs embedded in the loci of *Drosophila* NP and NPR genes using the REDfly database [37, 62]. Frequency and the length of these elements may influence the expression of their relevant genes [63]. To evaluate such parameters for NP and NPR genes, analyses in REDfly were performed for both unpaired and paired sets of NP and NPR genes, and results showed a significant increase in both quantity (number) and total length (kb) of CRMs in NPR genes versus NP genes (Fig. 1D,E). However, there were no significant differences in the average values of CRM lengths between the paired set of NP and NPR genes (Fig. 1F). These results suggest that the diversity and abundance of CRMs, rather than their individual lengths, are crucial for the transcriptional regulation of NPR genes. ReMap analysis [42] of five well‐characterized NP/NPR pairs in the *D. melanogaster* genome also supports the NPR‐biased enrichment of regulatory elements including TFBS in these loci, showing more diverse regulatory elements in the genomic regions of NPRs versus NPs (Fig. 1G–I). No significant regulatory element enrichment was found for a control group comprising *stan* (*CG11895*), *cirl* (*CG8639*), *mayo* (*CG11318*), *Remo* (*CG15744*), and *CG15556* genes, a class of adhesion G‐protein coupled receptors (aGPCRs), supporting the specificity of the enrichment for NPRs (Fig. S1A).

However, our analysis revealed a similar distribution pattern of NP and NPR gene expression across *Drosophila* tissues, as annotated in the *D. melanogaster* atlas (Fig. S1B). This similarity suggests that there is no significant difference in the diversity of tissues expressing NPs and NPRs. The equivalent tissue expression profiles indicate that the transcriptional regulation of NPRs may have evolved to emphasize temporal modulation rather than spatial specificity. This conclusion is based on the idea that comparable expression patterns across NP and NPR genes require a regulatory strategy capable of adapting to dynamic, context‐dependent changes in gene expression rather than being limited to spatial constraints.

### Spatial expression profile of NP and NPR genes across various tissues of *Drosophila melanogaster*

**Fig. 2:**
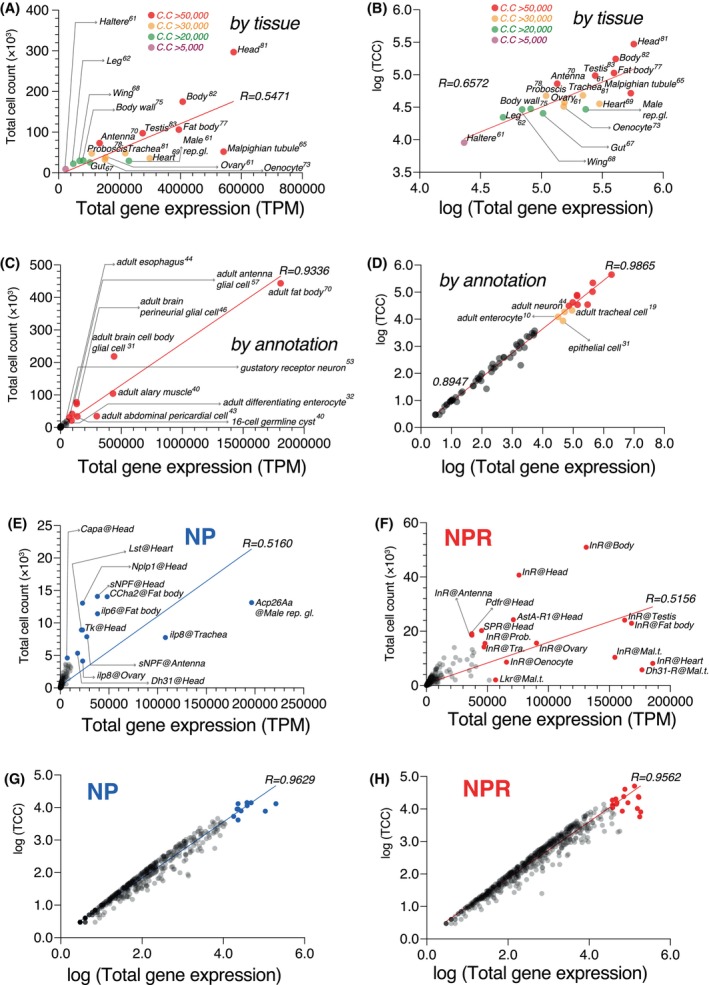
Spatial expression profiles and correlation analysis of NP and NPR genes across *D. melanogaster* tissues. (A, B) The scatter plot comparing the total gene expression, based on the transcript per million mapped reads (TPM) scale versus total cell count (TCC) in different tissues of *D. melanogaster*, analyzed by the Fly Cell Atlas single‐cell RNA‐seq pipeline (FCA; https://flycellatlas.org/), highlighting the head as a top‐ranked tissue with the highest level of gene expression compared to other tissues. Tissues are color‐coded by cell count (C. C) threshold. The correlation coefficient values (*R*) indicate a modest correlation between TCC and TPM when single cells are segregated by tissue types. Numbers on top of the name of the tissues denote the number of NPR and NP genes with detectable expression in each tissue. (C, D) Strong linear relationship between TPM and TCC values once the data are segregated by annotations in *D. melanogaster*. Numbers on top of each annotation denote the number of NPR and NP genes expressed in that annotated tissue cluster. (E, F) Scatter plots of TPM versus TCC, highlighting representative high‐expression NP (E) and NPR (F) genes, underscoring the role of NP‐NPR signaling in essential functions. Notably, *Acp26A* shows high expression in the male reproductive gland, while *sNPF*, *Nplp1*, *Tk*, *Dh31*, and *Capa* are predominantly expressed in the head. *InR* is the most abundantly expressed receptor, particularly in the body and heart, emphasizing its role in systemic signaling. (G, H) Strong linear relationship between TPM and TCC for both NP and NPR genes across different tissues in *D. melanogaster*.

First, we analyzed NPR gene expression levels across various tissues of *D. melanogaster* using the most recent scRNA‐seq data in the Fly Cell Atlas [28]. The head region showed the highest NPR expression level, presented as transcripts per million mapped read (TPM) [64] (Fig. 2A), consistent with its importance in the central nervous system (CNS) and in relationship with NP signaling processes. A modest correlation was found between gene expression levels and the number of cells expressing these genes in the respective tissues (Fig. 2B, *R* = 0.6572), suggesting additional layers of regulatory mechanisms. When we categorized the expression pattern of NPRs by tissue function, a strong linear relationship emerged between NPR gene expression and cell counts (Fig. 2C,D, *R* = 0.9865), indicating a closer link between NPR activity and cell numbers when considering tissue functionality rather than the physical architecture of tissues. These findings also highlight the *Drosophila* head as a critical area for NP‐NPR signaling and suggest complex regulatory mechanisms governing NPR expression.

We also examined the expression profile of individual NPs and NPRs across various *Drosophila* tissues (Fig. 2E,F). *Acp26A* was notably abundant in the male reproductive gland, suggesting its role in reproduction. Other NPs such as *sNPF*, *Nplp1*, *Tk*, *Dh31*, and *Capa* were primarily expressed in the head, while *ilp6* [65] and *CCHa2* [66, 67, 68] were active in the fat body, associated with energy metabolism. The expression of *sNPF* in the antenna aligns with previous findings [69] and supports our approach. In comparison with NPs, NPRs exhibited different levels of gene expression in the examined tissues, with *InR* (which binds to multiple insulin‐like peptides [70]) being the most prevalent gene in the *D. melanogaster* tissues (Fig. 2F). Its high expression in the body and heart highlights the significance of NP‐NPR signaling for vital functions. We observed a strong positive correlation between gene expression levels (TPM) and the number of expressing cells (TCC) for both NPs and NPRs, indicating that tissues with high expression of NP and NPR genes harbor more cells expressing these genes (Fig. 2G,H). Altogether, these findings suggest that rather than tissue types, tissue functionality and/or cell type varieties are correlated with NP/NPR expression levels (Fig. S2). In fact, while no distinct “tissue specificity” was found for the NP and NPR genes, their “expression levels” differ across the tissues based on the differences in the functionality, developmental and/or physiological states, or cell type composition of the tissues.

### Landscape of *drosophila* NP‐NPR‐TF network

To investigate the network of transcription factors (TFs) regulating the expression of NP and NPR genes in context‐dependent behaviors, we focused on 10 NPs and 13 NPRs linked to interval timing behaviors (Table 2). This selection supports our aim to examine how a limited set of NP‐NPR pairs modulates complex behaviors, as shown in previous studies [52, 71, 72, 73]. Through applying the AUCell algorithm [47, 74] on the scRNA‐seq data available in the Fly Cell Atlas, the interactome profile of NP and NPR genes with their transcription factors (TFs) was visualized, revealing the transcriptional mechanisms that shape the NP‐NPR network architecture.

**Fig. 3:**
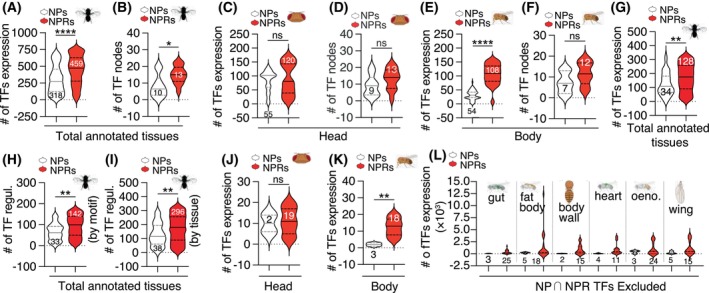
The relationship between transcription factors (TFs) that regulate the transcription of NP and NPR genes. (A, B) Number of TFs and TF nodes regulating NP and NPR genes across all annotated tissues of *D. melanogaster*. (C, D) Number of TFs and TF nodes regulating NP and NPR genes in the *Drosophila* head. (E, F) Number of TFs and TF nodes regulating NP and NPR genes in the *Drosophila* body. (G) Number of TFs regulating NP and NPR genes, after removal of common (shared) TFs expressed in both NP‐ and NPR‐expressing cells. (H, I) Number of NP‐ and NPR‐related regulons, when the TFs common between the NP‐ and NPR‐expressing cells are removed. Regulons are classified and counted based on their motifs (H) and tissues (I). (J, K) Number of TFs regulating NP and NPR genes in the *Drosophila* head (J) and body (K), once the TFs shared between the NP‐ and NPR‐expressing cells are excluded. (L) Number of TFs regulating NP and NPR genes in different *D. melanogaster* tissues, after removal of common TFs of NP‐ and NPR‐expressing cells. Statistical significance of the graphs was evaluated using unpaired t‐test. Statistically significant and non‐significant differences are denoted as asterisks (*P < 0.05, **P < 0.01, ***P < 0.001, ****P < 0.0001) and ‘ns’ (*P* > 0.05), respectively.

Our analysis identified distinct patterns of TF interaction for NP and NPR genes, in which the cells expressing NPRs showed significantly more interacting TFs expressed (Fig. 3A). Also, the NPR‐associated TFs demonstrated greater connectivity than those linked to NPs (Fig. 3B), indicating more complex transcriptional regulation governing NPR gene expression (Fig. S3A).

In the *Drosophila* head tissue, the dominance of NPRs in association with TF networks diminished. Although interacting TFs were higher in the NPR‐expressing cells compared to the NP‐expressing cells, the differences were not significant in the *Drosophila* head (Fig. 3C). Similarly, the node counts remained unchanged across the cells expressing NP and NPR genes (Fig. 3D), suggesting similar levels of transcriptional control for both classes of genes in this tissue. Unlike the similarities in the TF networks linked to NP and NPR genes in the *Drosophila* head (Fig. 3C,D, Fig. S3B), the NPR‐TF network in the body was significantly more complex than the NP‐TF network, with a greater number of TFs in NPR‐expressing cells rather than NP‐expressing cells (Fig. 3E, Fig. S3C), though node disparities were not statistically significant (Fig. 3F).

These findings highlight the longstanding question regarding the diverse roles of NPs, which often function as hormones. Unlike neurotransmitters that primarily act within synapses, NPs influence distant targets through targeting the receptors expressed throughout the body, including peripheral tissues. In humans, NPs can modulate synaptic transmission efficacy by enhancing or inhibiting co‐released neurotransmitters and can act like peptide hormones to affect various physiological functions [75]. Given that NPRs serve as receptors for both NPs and peptide hormones, it is unsurprising that the NPR‐TF network is more complex in body tissues than in head tissues. In *D. melanogaster*, neuropeptides like *AstA* and *NPF* are expressed in both brain and gut and act bifunctionally as neuropeptides and/or hormones through shared NPRs [76, 77, 78]. This complexity likely reflects diverse physiological demands across body systems compared to the head.

Excluding the TFs shared between the NP‐ and NPR‐expressing cells revealed clearer network patterns for NP‐TF and NPR‐TF interactions (Fig. S3D). The removal of common TFs indicated a persistent bias toward NPRs, suggesting that NPR‐expressing cells engage in a more complex TF network (Fig. 3G). The cascade of TFs is crucial for controlling developmental processes [79] and generating diversity in enteroendocrine cells [80]. Furthermore, TF cascades are vital for neuronal diversity [81], synaptic plasticity [82], and long‐term memory formation [83]. As anticipated, TFs regulated by other TFs were significantly more prevalent in NPR‐expressing cells compared to NP‐expressing cells, both in network motifs and tissue context (Fig. 3H,I). These results suggest that TFs exclusive to NPR‐expressing cells are highly regulated by cascades that may control behavioral plasticity necessary for NP modulation of various context‐dependent behaviors.

When considering nonshared TFs between NP‐ and NPR‐expressing cells, both head and body networks showed a bias toward NPR‐expressing cells, particularly in the body (Fig. 3J–L; Fig. S3E,F), supporting our earlier observations of more complex TF networks for NPRs versus NPs, particularly in peripheral tissues. After excluding common TFs shared between NP‐ and NPR‐expressing cells, we also identified six tissues (gut, fat body, body wall, heart, oenocyte, and wing) where NP‐expressing cells had unique TFs. In these tissues, NPR‐expressing cells had higher TF numbers and expression levels (Fig. 3L), suggesting that NP‐expressing cells primarily rely on shared TFs used by NPR‐expressing cells across most tissues.

### Tissue‐specific biases in the regulatory networks of NPs and NPRs

We assessed the NP and NPR regulatory networks across various tissues, focusing on internal organs including Head, Body, Testis, Antenna, Body wall, Fat body, Gut, Haltere, Heart, Leg, Male reproductive glands, Malpighian tubule, Oenocyte, Ovary, proboscis, and maxillary palps, Trachea, and Wing, where transcriptional regulation is crucial for metabolism and infection responses [84, 85, 86, 87, 88, 89]. Across all the examined internal organs, NPR‐expressing cells exhibited a greater number of expressed interacting TFs than NP‐expressing cells, with significant differences (Fig. S4) except in the trachea (Fig. S4P–R).

We also investigated transcriptional control in sensory organs, known for their high level of gene expression regulation (Fig. S5) [90]. Most sensory organs showed an increased number of TFs in the interaction network of NPR‐expressing cells, except in the antenna, where NP‐expressing cells had higher TF levels (Fig. S5A–C). This might be due to the abundant expression of the *short neuropeptide F* (*sNPF*) in the antennal lobe, essential for olfactory function [69, 91].

In sexually dimorphic organs (Fig. S6), we found higher TF expression in NP‐expressing cells of the male reproductive gland (Fig. S6A–C), likely linked to accessory gland proteins (Acps) affecting female physiology [92, 93, 94]. *Acp26a* was highly expressed in this gland (Fig. 2E), but overall TF networks were richer in NPR‐expressing cells (Fig. S6D,E). The female ovary and male testis followed the trend seen in other tissues, indicating that NP‐biased TF networks in the antenna and male reproductive organ are exceptions (Fig. S6).

### Transcriptional regulation of NP signaling during aging of *Drosophila melanogaster*

Aging is a biological process marked by declining physiological function and increased vulnerability to age‐related diseases. At the molecular level, aging involves remarkable changes in gene expression driven by transcriptional control mechanisms. TFs are crucial components in this process and bind to specific DNA sequences to regulate the genes involved in cellular maintenance, stress response, and metabolism [95]. The transcriptional regulation of aging is influenced by various TFs and signaling pathways, particularly those in the insulin/insulin‐like growth factor 1 signaling (IIS) pathway, the target of rapamycin (TOR) pathway, and the FOXO family of TFs. These factors affect lifespan and health span by regulating genes related to antioxidant defense, DNA repair, autophagy, and mitochondrial function [96, 97, 98].

**Fig. 4:**
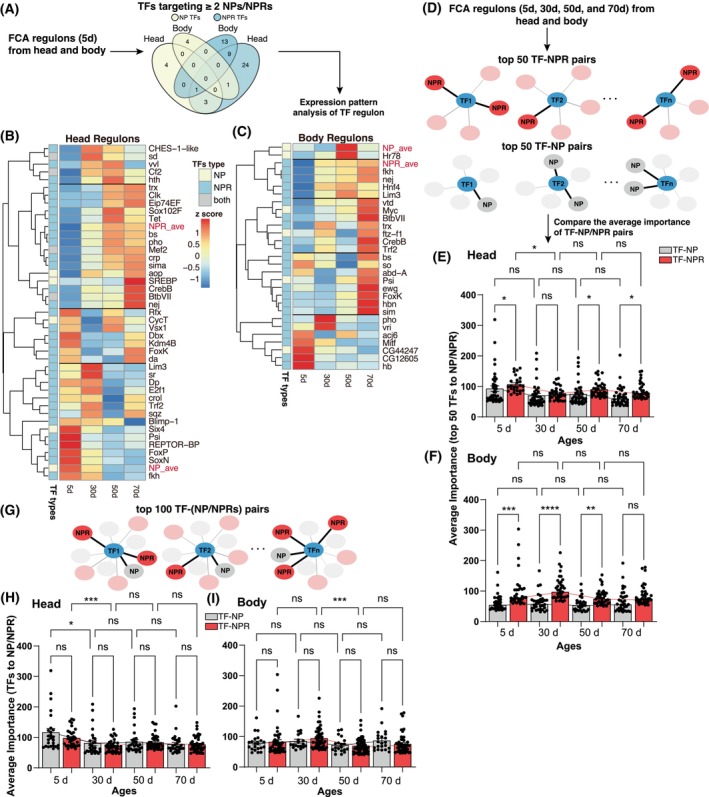
Identification and analysis of transcription factors (TFs) regulating the expression of neuropeptide (NP) and neuropeptide receptor (NPR) genes during aging in *D. melanogaster*. (A–C) Identification of TFs involved in the regulation of NP and NPR genes across different ages and a schematic overview of the process for identifying TFs that regulate NP and NPR genes using regulon data from the Aging Fly Cell Atlas (AFCA). The Venn diagram (A) illustrates the overlap between the number of TFs targeting NP and NPR genes in the *Drosophila* head and body, showing more TFs in association with NPRs rather than NPs, with a minimal overlap between them. (B, C) Heatmaps illustrate the average expression level of NP‐ and NPR‐related regulons across various developmental time points in the *Drosophila* head and body, revealing distinct age‐related patterns in the regulation of NP and NPR genes by TFs. (D–F) Analysis of the significance of TF‐NP and TF‐NPR regulatory pairs across different developmental time points of *D. melanogaster*. The overall view of the approach used to rank and compare the top 50 TF‐NP and TF‐NPR pairs based on their importance scores is illustrated for the time points (D). Bar plots show the comparisons between the average importance of top TF‐NP and TF‐NPR pairs in the head (E) and the body (F) across indicated developmental time points (ages), showing that TF‐NPR pairs (red trend lines) generally have higher importance scores than TF‐NP pairs (black trend lines) across these ages. (G–I) Examination of the top 100 TF‐NP and TF‐NPR pairs. Network diagram (G) represents the top 100 TF‐NP and TF‐NPR regulatory pairs, with TF‐NPR pairs consistently outnumbering TF‐NP pairs, suggesting a more complex regulatory network for NPR genes. Bar plots (H, I) show the average importance of TF‐NP and TF‐NPR pairs across different ages in the *Drosophila* head and body, with results indicating that although NPR genes are regulated by more TFs, the overall importance scores for these pairs do not significantly differ between NP and NPR networks as age progresses. For the graphs, the statistically significant and non‐significant differences are denoted as asterisks (*P < 0.05, **P < 0.01, ***P < 0.001, ****P < 0.0001) and “ns” (*P* > 0.05), respectively.

Based on our findings that transcriptional control is biased toward NPR‐expressing cells, we examined age‐related changes in adult *D. melanogaster*. Using regulon data from the Aging Fly Cell Atlas (AFCA) [45], we identified several TFs regulating both NP and NPR genes across different ages in the head and body. More TFs were found to regulate NPRs than NPs, with minimal overlap between the two (Fig. 4A). At the RNA level, NPR expression increases with age, while NP expression peaks at 5 days in the head and declines thereafter. In the body, NP expression is highest at 50 days (Fig. 4B,C). Additionally, slightly more regulons co‐express with NPRs than with NPs as flies age (Fig. 4A–C). Our data illustrate differential expression patterns for NP and NPR genes and their associated regulons.

To explore differences in the regulatory networks associated with NPs and NPRs during aging, we analyzed TF‐gene networks at each developmental time point. We ranked TF‐NP and TF‐NPR pairs by importance and selected the top 50 pairs for comparison (Fig. 4D). In the head, the importance of TF‐NP pairs slightly decreases with age, while TF‐NPR pairs show a more pronounced decline. At many time points, TF‐NPR pairs exhibited significantly more importance than TF‐NP pairs (Fig. 4E). In the body, the differences between TF‐NP and TF‐NPR pairs are more pronounced, in which TF‐NPR pairs show significantly higher importance at 5, 30, and 50 days but a slight increase at 70 days, while TF‐NP pairs remain relatively constant without clear trends (Fig. 4F). These findings indicate that top‐ranked TF‐gene pairs reveal clearer regulatory distinctions between NPR and NP genes.

To further address the cell type‐specific aspects of transcriptional regulation during aging, we examined the regulatory relationships across several key neuronal types—including antennal lobe projection neurons, dopaminergic PAM neurons, gustatory receptor neurons, Kenyon cells, octopaminergic/tyraminergic neurons, and olfactory receptor neurons—at different ages in adult *D. melanogaster*.

When comparing the top 50 TF‐NP and TF‐NPR pairs by importance score within each cell type, we observed distinct regulatory trends. In antennal lobe projection neurons, gustatory receptor neurons, and olfactory receptor neurons, TF‐NPR pairs consistently exhibited significantly higher importance than TF‐NP pairs across almost all time points (Fig. S7A,C,F). In Kenyon cells and dopaminergic PAM neurons, TF‐NPR dominance was observed at early and late ages (5d and 70d), with no significant differences at mid‐life stages (Fig. S7B,D). Interestingly, in octopaminergic/tyraminergic neurons, TF‐NPR pairs were more important at 30 days, while TF‐NP pairs gained higher importance at 50 days, suggesting a dynamic shift in regulatory priorities during aging (Fig. S7E).

We further expanded this analysis by pooling all TF‐NP and TF‐NPR pairs and selecting the top 100 ranked interactions by importance. In contrast to the top 50 comparison, the aggregated top 100 analysis revealed no significant differences in regulatory strength between TF‐NP and TF‐NPR pairs across most cell types and ages (Fig. S7G–L). This indicates that while NPR genes are regulated by a larger number of TFs overall, the average regulatory intensity may not differ markedly from that of NPs when considering a broader range of interactions.

Taken together, these results underscore that transcriptional control of NPR genes is not only more extensive—involving more TFs—but also more cell type‐ and context‐dependent, especially in neurons involved in sensory perception and neuromodulation. The stronger and more selective TF‐NPR interactions observed in top‐ranked pairs suggest that NPR genes are subject to tighter transcriptional regulation by high‐importance TFs, potentially reflecting their pivotal roles in age‐related physiological adaptation in *Drosophila melanogaster*.

Expanding our analysis to the top 100 TF‐NP/NPR pairs for the whole body and head (Fig. 4G), we consistently found more TF‐NPR pairs than TF‐NP pairs across all ages; though importance scores did not significantly change as age increased (Fig. 4H,I). These data indicate that NPR genes are regulated by more TFs, suggesting a more complex regulatory network associated with NPRs versus NPs.

In conclusion, compared to the NP genes, NPR genes are involved in regulatory networks with more TFs, while individual regulatory intensity is similar for both NPR and NP genes at a general level. Differences in top‐ranked pair importance suggest that NPR genes are influenced more by key TFs with strong regulatory effects, while NP gene regulation appears broader but less concentrated on high‐importance TFs.

Our findings indicate that TF‐NPR networks are significant in aging *D. melanogaster*. To determine whether transcriptional control is also crucial for NP‐NPR signaling during early adult development, we utilized scRNA‐seq data from 1‐day‐ and 3‐day‐old adult flies compared to the aforementioned analyzed networks of 5‐day‐old adults (Fig. 4A–C) [28]. Results showed that the number of cells expressing NPs peaked at Day 3; then decreased after this time, while the number of cells expressing NPRs was significantly increased and continued to rise through Day 5 (Fig. S7M). Network complexity was indistinguishable in 1‐day‐old adults (Fig. S7N–P), while NPR‐TF network complexity significantly increased through Day 3 with elevated TF expression observed (Fig. S7Q–S). A significant increase in the TF numbers in networks of both NP and NPR genes occurred between Days 3 and 5 (Fig. S7T). These findings support that during early adult development from Day 1 to Day 5, encompassing sexual maturation, NP‐NPR signaling is highly regulated by TF networks, particularly favoring NPR‐TF interactions.

### Transcriptional regulation of NP‐NPR pairs in *Drosophila melanogaster*

Our study reveals that diverse transcriptional regulation of NPR expression mediated by different combinations of NP‐NPR pairs influences context‐dependent behaviors. Global patterns indicate tight transcriptional control mediated by TFs in NPR‐expressing cells wherein each NP‐NPR pair is regulated by a unique TF. Next, we focused on the insulin signaling pathway, a well‐studied system crucial for metabolic balance, aging, and diseases [86, 87, 88, 99]. In *D. melanogaster*, the insulin signaling—which is under the control of insulin receptors (InRs) and other signaling pathways like PI3K/AKT/FOXO—can modulate metabolism, growth, and lifespan [100, 101, 102, 103, 104].

Comparative analysis of TF expression profile between *ilp*‐ and *InR*‐expressing cells revealed a higher expression rate of TFs in *InR*‐positive cells, highlighting stronger transcriptional control in NPR‐expressing populations (Fig. S8A–E). Most NP‐NPR pairs exhibited NPR‐biased TF regulation, similar to *ilp‐InR* pair, though exceptions such as *AstA*‐*AstA‐R2* showed NP‐biased control (Fig. S8H,I). AstA‐R1 regulates developmental maturation [105, 106], while AstA‐R2 mediates sleep‐deprivation‐induced energy wasting [76, 107]. *AstC*‐*AstC‐R2* displayed head‐specific TF regulation (Fig. S8L,M), contrasting with body‐biased control in *sNPF*‐*sNPF‐R* and *Capa*‐*CapaR* pairs (Fig. S8N–Q).

*CCHa1*‐*CCHa1‐R* exhibited NPR‐biased regulation, while *CCHa2*‐*CCHa2‐R* showed NP‐biased control (Fig. S9), reflecting distinct roles in sleep and appetite [66, 67]. The diuretic hormones Dh44 and Dh31, involved in fluid balance and circadian regulation, showed limited TF control in receptor‐expressing cells [108, 109, 110, 111, 112, 113]. Tachykinin (Tk) signaling, involving TkR99D and TkR86C, displayed NP‐biased regulation, contrasting with natalisin's receptor‐biased control [114, 115, 116, 117]. *Hugin*‐*PK2*‐*R1/R2* pairs showed head‐biased NP regulation and body‐biased NPR control, reflecting roles in growth and feeding [118, 119, 120, 121, 122]. *Myosuppressin* (*Ms*)‐*MsR1* exhibited NPR‐biased regulation, while *MsR2* lacked TF control in the head [123] (Fig. S10).

Insulin‐like peptides *ilp7*‐*Lgr4* and *ilp8*‐*Lgr3* displayed NPR‐ and NP‐biased regulation, respectively (Fig. S10O–R), aligning with their roles in development and anorexia [100, 124, 125, 126, 127, 128]. *Proctolin* (*Proc*)‐*ProcR* showed head‐biased NP and body‐biased NPR regulation, consistent with its role in muscle contraction [108]. *Nplp1*‐*Gyc76C*, involved in immune response, exhibited NP‐biased regulation [129] (Fig. S11A–D). Other pairs, including *Trissin*‐*TrissinR*, *Pdf*‐*Pdfr*, and *SIFa*‐*SIFaR*, consistently demonstrated NPR‐biased regulation (Fig. S11E–P), highlighting the predominance of stringent transcriptional control in NPR‐expressing cells across diverse NP‐NPR systems. This observation implies that across various NP‐NPR pairs, despite utilization of distinct transcriptional control mechanisms, the cells expressing NPRs are predominantly under stringent transcriptional control in most cases.

### Empirical validation of NPR‐biased transcriptional regulation

To investigate whether the NPR‐biased transcriptional regulation is influenced by environmental changes, qRT‐PCR was performed on a set of NP‐NPR pairs including *AstA*‐*AstA‐R1*/*R2*, *Crz*‐*CrzR, SIFa*‐*SIFaR*, and *sNPF*‐*sNPF‐R*. These pairs were chosen due to their roles in integrating neuropeptide inputs, circuit‐specific signaling, and hormone‐mediated state‐dependent responses [71, 73, 114, 121, 130]. We applied temperature shifts as an environmental stimulus known to induce significant behavioral and physiological changes in *Drosophila* [131, 132, 133]. Thereafter, the expression profile of the NP‐NPR pairs was investigated separately in the dissected head and body tissues of both female and male flies with different ages (1, 3 and 5 days post eclosion).

**Fig. 5:**
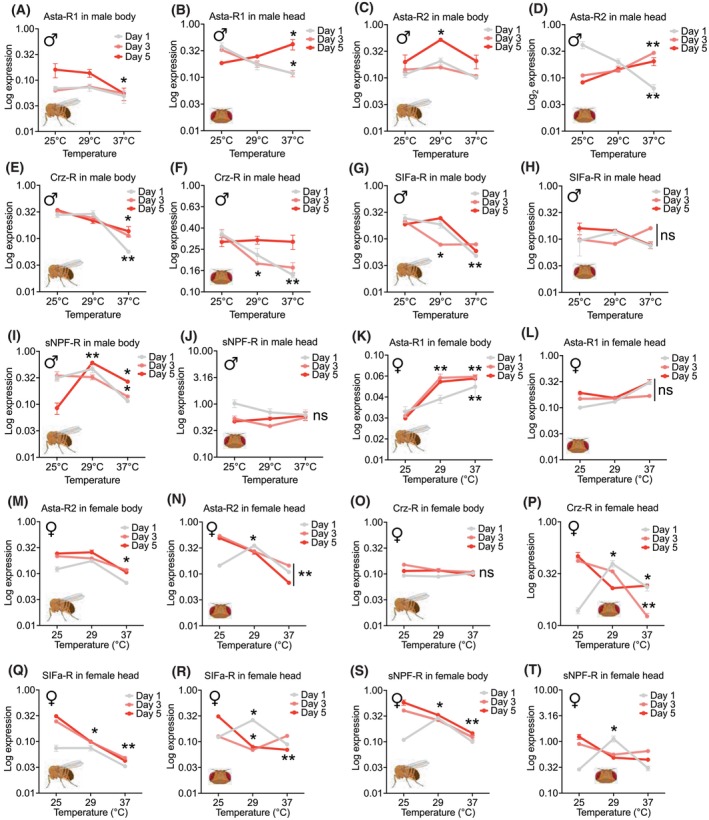
Expression changes of neuropeptide receptor (NPR) mRNAs in *D. melanogaster* under different temperature conditions. (A–J) Expression level of NPR mRNAs (normalized to the *GAPDH* gene expression level) in the dissected “body” and “head” tissues of male flies with different ages (1, 3, and 3 days after eclosion). Results are represented as the mean ± SEM from three independent biological replicates. Unpaired t‐test was used to evaluate the statistical significance of the data (*P < 0.05 and **P < 0.01). Nonsignificant changes are indicated by “ns.” (K–T) Expression level of NPR mRNAs (normalized to the *GAPDH* gene expression level) in the dissected “body” and “head” tissues of female flies with different ages (1, 3, and 3 days after eclosion). Results are represented as the mean ± SEM from three independent biological replicates. Unpaired *t*‐test was used to evaluate the statistical significance of the data (*P < 0.05 and **P < 0.01). Nonsignificant changes are indicated by “ns.”

**Fig. 6:**
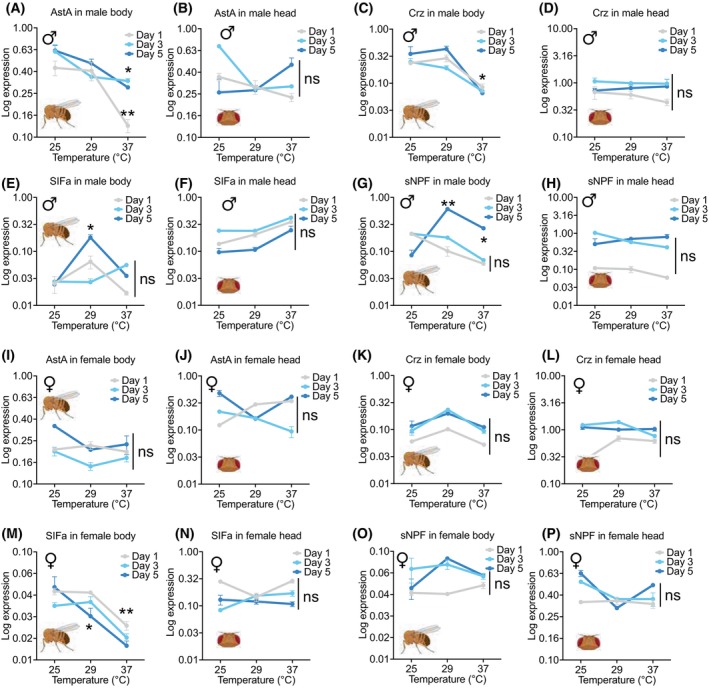
Expression changes of neuropeptide (NP) mRNAs in *D. melanogaster* under different temperature conditions. (A–H) Expression level of NP mRNAs (normalized to *GAPDH* gene expression) in the “body” and “head” tissues of male flies at different ages (1, 3, and 3 days after eclosion). Results are represented as the mean ± SEM of three independent biological replicates. Unpaired *t*‐test was used for evaluation of the statistical significance of the data (*P < 0.05 and **P < 0.01). Non‐significant changes are indicated by “ns.” (I–P) Expression level of NP mRNAs (normalized to the *GAPDH* gene expression level) in the “body” and “head” tissues of female flies with different ages (1, 3, and 3 days after eclosion). Results are represented as the mean ± SEM of three independent biological replicates. Unpaired t‐test was used for evaluation of the statistical significance of the data (*P < 0.05 and **P < 0.01). Non‐significant changes are indicated by “ns.”

Results showed that, while mRNA levels of NPs are mostly stable during temperature shifts, mRNAs of NPR genes were significantly increased or decreased when the temperature is high, implying a crucial role of NPR transcriptional control under this environmental change (Figs 5 and 6). In fact, the expression level of all the studied NPRs, including *AstA‐R1*, *AstA‐R2*, *Crz‐R*, *SIFa‐R*, and *sNPF‐R*, was changed when flies were exposed to higher temperatures (29 °C and 37 °C) compared to the flies living all the time in normal conditions (25 °C, room temperature) (Fig. 5). In contrast, the expression level of ligands of these NPRs, including *AstA*, *Crz*, *SIF*, and *sNPF*, was relatively unchanged, particularly in the *Drosophila* head tissue, wherein all the examined NPs did not exhibit any significant change in their expression (Fig. 6). Consistently, the expression level of NPR genes exhibited a meaningful expression pattern (decreased or increased) throughout temperature shifting, unlike NP genes, which exhibited a scattered expression pattern during the same condition (Fig. S12). These findings support the hypothesis that NPR‐biased transcriptional regulation is an indispensable mechanism for modulating context‐dependent behaviors in *D. melanogaster*. Also, these findings provide strong empirical support for our central hypothesis: NPRs are more dynamically regulated than NPs in response to environmental and physiological cues. This asymmetric regulatory flexibility likely endows the neuropeptidergic system with the capacity to fine‐tune behavioral and metabolic outputs, enabling context‐dependent adaptation through selective receptor‐level modulation.

While a greater number of TFs are found to regulate NPR genes, our analysis of TF‐NP and TF‐NPR networks also showed a set of TFs that could regulate the expression of NP and NPR genes simultaneously (Tables S1 and S2). Table S1 lists these interactions segregated by their annotations (cell types) with their frequencies in each annotation (transcript counts) (Table S1). Table S2 represents the interactions of such common TFs disregarding classification based on annotations and/or cell types, but emphasizing the regulon information of each TF (Table S2). The network of these common TFs with 10 representative NP‐NPR pairs is also illustrated in Figs S13 and S14, which suggest the existence of a more complex network in the *D. melanogaster* head for the common TFs, in comparison with the networks of *D. melanogaster* body (Figs S13 and S14).

## Discussion

Ability to sense, remember, anticipate, or act emerges from the way that the nervous system of an organism is organized into networks that allow signals to flow, interact, and change [134]. A crucial component of signaling molecules active in this system is neuropeptides and their relevant signaling pathways whose signals are originated from a particular set of cells and scattered throughout the body to bind to their receptors called neuropeptide receptors (NPRs). While the expression pattern and biological function of many NPRs were vastly studied, the mechanisms underlying their regulation and how they respond to environmental changes remain obscure.

This study explored how transcriptional regulation of NPRs modulates context‐dependent behaviors and physiological responses in *Drosophila melanogaster*. The availability of extensive genetic tools and resources for studying *D. melanogaster*, such as chemiconnectome profiles of the fly [135], and the tools for studying them (i.e., chemiconnectomics [136] enable us to unravel the mechanisms underlying the link between the *Drosophila* nervous system function and context‐dependent behaviors [137]).

Using comparative genomics, expression patterns, and transcription factor (TF) network analysis, together with empirical validation, we showed that NPR gene expression is tightly regulated by environmental and physiological cues, underscoring the critical role of NPR expression in neuropeptidergic signaling. We also showed that NPR genes exhibited harboring more complex *cis*‐regulatory landscapes, with more enhancers than NP genes, suggesting their greater potential for regulation by upstream signals or processes. Transcription factor binding site (TFBS) HOT spots are genomic regions with high potential for occupancy with transcription factors (TFs). In this study, we analyzed both “frequency” and “length” features of these elements for both NP and NPR genes, in which NPR genes exhibited longer sequences and greater numbers of TFBS‐HOT spots consistent with their tighter regulation by upstream factors. These elements can be located on any genomic region of NPR genes including intergenic regions (e.g., close to promoters), intronic spaces, and, to a lesser extent, within the exons (e.g., UTRs or coding sequences), and confer more complex regulatory potential to NPR genes rather than their ligands (NPs). Consistent with the higher frequency of TFBS HOT elements in NPRs versus NPs, the CRMs—whose regulatory influences are mostly experimentally validated through tests such as *in vivo* reporter genes and cell culture assays—are also presented more frequently in the genomic regions of NPRs versus NPs (Fig. 1). Similarly, CRMs can be distributed in any location of genes [63].

In addition to possessing a more complex genomic regulatory landscape, NPRs are also under regulation by more TFs, indicating a robust transcriptional network (Fig. 3). We showed that NPR expression increases with *Drosophila* age, with stronger TF regulation compared to NPs, suggesting a key role in aging (Fig. 4). These findings are in line with the concept that NPR genes are in different locations or in different amounts during development and/or physiological changes of an organism. More complex genomic regulatory elements in NPR genes together with their diverse TF networkscould potentially mediate the transcriptional regulation of NPRs, which are ubiquitously expressed, compared to the narrower expression pattern of NPs [136, 138].

In addition to the increased frequency and wider dispersion of TFBS‐HOT spots and CRMs in NPR genomic loci, cooperative activity of TFs at their genomic regions may also play a crucial role for spatiotemporal expression control of NPRs. Therefore, in this study, the interactome profiles of TFs interacting with NPs (TF‐NP networks) and NPRs (TF‐NPR networks) were investigated. We showed that, during early adult development, TF‐NPR interactions dominate, particularly during sexual maturation (Figs S6, S7). Differential transcriptional regulation of NP‐NPR pairs reveals context‐specific control mechanisms, in which the majority of NP‐NPR pairs exhibit NPR‐biased while the minority exhibit NP‐biased patterns of regulation (Fig. S8–S11). Empirical validation through qRT‐PCR confirmed that in most cases, NPR expression, but not NP levels, responds to temperature shifts, emphasizing NPRs' role in adaptive signaling. Consistent with our findings, the transcription level of NPR genes was previously found to be affected by demographic features like sex, age, and environmental influences [139]. We also showed that expression changes of NPR genes upon temperature shifts could be affected by age and sex in *D. melanogaster* (Figs 5 and 6; Fig. S12). For instance, such expression changes may differ between newly eclosed flies and adult ones, so that some NPRs such as *Asta‐R2* exhibited differential expression changes in 1‐day‐old flies versus adult flies in which its expression significantly decreased in 1‐day‐old flies while increased in the 3‐ and 5‐day‐old flies (Fig. 5D), suggesting crucial effects of the age factor on NPR‐mediated responses. In addition, the pattern of expression changes may differ by sex. For instance, *sNPF‐R* (an NPR) is significantly downregulated at higher temperature in females while its expression level increases under these conditions (Fig. S12A,B vs. E,F). These findings altogether confirm differential responses of NPR genes during environmental changes, which are also affected by other intrinsic factors of an organism such as developmental time and sex.

Transcriptional regulation of NPR gene expression might not be the sole mechanism involved in context‐dependent adaptation, and other post‐transcriptional events such as alternative splicing (AS) may also play roles to change the expression level of NPs and NPRs [140, 141]. Since the prevalence of AS was reported to be equal (in general) for NP and NPR genes [142], the observed differential expression patterns of NPR genes under physiological and/or environmental changes could not be due to the distinctive pattern of AS in NPR transcripts. Such a regulatory landscape was previously confirmed for the odor receptor genes, where their expression pattern was shown to be determined primarily by the presence of upstream and downstream genomic elements, rather than AS. In fact, in the *Drosophila* olfactory system, receptor gene expression is primarily driven by combinatorial codes of *cis*‐acting genomic elements that recruit different TFs for transcriptional control of the genes [143]. Instead, AS acts primarily to diversify the transcript products of a gene as well as cell type switching rather than expression level changes [140]. Consistently, during an ‘exitron splicing’ event observed in the *Drosophila* odor receptor genes, the transcriptional levels of all alternatively spliced variants were found to be similar, and AS only diminished the coding potentiality of the mRNAs encoded by these genes [144].

Therefore, while both transcriptional and post‐transcriptional regulatory mechanisms could change the expression pattern of NP and NPR genes, under environmental changes and/or exposure to extracellular stimuli, the NPR‐biased regulatory mechanisms are mostly related to their differential connection with TFs and also due to possessing different TFBS and CRM profiles.

Given the evident roles of NP and NPR genes in context‐dependent adaptation processes [145, 146, 147, 148], the findings of this study will help scientists to narrow down the broad lists of NP‐NPR pairs into shorter lists of candidates to find the NP‐NPR pairs with the most likely functionality in any physiological and/or behavioral process. For instance, in the head tissue, the annotations of cells that highly express TFs regulating NP transcription are as follows: T neuron T4/T5c‐d, adult brain cell body glial cell, and photoreceptor cell R8, in this order. The TFs that are most involved in NP transcription regulation are *CG44247*, *dim*, and *hth*. The NPs with the highest expression are *sNPF*, *AstA*, and *FMRFa*.

In the head tissue, the annotations of cells that highly express TFs regulating NPR transcription are as follows: T neuron T4/T5c‐d, transmedullary neuron Tm20, and proximal medullary amacrine neuron Pm2, in this order. The TFs most involved in NPR transcription regulation are *hth*, *sr*, and *Mef2*. The NPRs with the highest expression are *SIFa‐R*, *AstA‐R1*, and *sNPF‐R*.

In the head tissue, the annotations of cells that highly express TFs co‐regulating both NP and NPR transcription are as follows: olfactory receptor neuron, adult brain perineurial glial cell, and adult reticular neuropil associated glial cell, in this order. The TFs most involved in coregulating NP and NPR transcription are *hth*, *CG44247*, and *sr*. The NP/NPR pairs with the highest expression are *sNPF*/*sNPF‐R*, *AstA*/*AstA‐R1*, and *AstC*/*AstC‐R2*.

In the body tissue, the annotations of cells that highly express TFs regulating NP transcription are as follows: perineurial glial sheath, cell body glial cell, and prefollicle cell/stalk follicle cell, in this order. The TFs most involved in NP transcription regulation are *dim*, *CG44247*, and *sr*. The NPs with the highest expression are *natalisin*, *sNPF*, and *AstA*.

In the body tissue, the annotations of cells that highly express TFs regulating NPR transcription are as follows: indirect flight muscle, adult peripheral nervous system, and cell body glial cell, in this order. The TFs most involved in NPR transcription regulation are *Mef2*, *svp*, and *Trf2*. The NPRs with the highest expression are *Pdfr*, *sNPF‐R*, and *Crz‐R*.

In the body tissue, the annotations of cells that highly express TFs co‐regulating both NP and NPR transcription are as follows: adult ventral nervous system, multidendritic neuron, and muscle cell, in this order. The TFs most involved in co‐regulating NP and NPR transcription are *sr*, *Atf6*, and *CG16779*. The NP/NPR pairs with the highest expression are *sNPF*/*sNPF‐R*, *AstA*/*AstA‐R1*, and *AstC*/*AstC‐R1*.

In comparison with the NP regulatory and/or interaction networks, the NPR‐biased regulatory factors are highlighted to be more involved in adaptation‐related processes. The importance of transcriptional regulation of NPR genes, as revealed by this study, has broad implications for our understanding of neural circuit function and plasticity. The ability to modulate NPR expression in response to environmental cues and physiological states allows for the dynamic adjustment of neuropeptidergic signaling, which may underlie the adaptability and complexity of behaviors and physiological processes. This regulatory strategy may be particularly important for survival and reproduction in a changing environment. Future research should identify specific TFs and pathways regulating NPR expression across tissues and conditions, with potential implications for human health and disease.

### Limitations of the study

While this study provides insights into the transcriptional regulation of NPRs, it has several limitations. The environmental conditions tested, such as temperature shifts, represent only a subset of potential stimuli, limiting the generalizability of the findings. Additionally, the qRT‐PCR validation was restricted to a few NP‐NPR pairs, leaving other interactions unexplored. The reliance on computational predictions for TF networks and *cis*‐regulatory elements, without extensive experimental validation, may overlook context‐specific regulatory mechanisms. Furthermore, the study focuses primarily on *D. melanogaster*, and while evolutionary conservation is suggested, direct applicability to other species, including mammals, requires further investigation. These limitations highlight the need for broader experimental conditions, expanded validation, and cross‐species studies to fully elucidate the regulatory landscape of neuropeptidergic signaling.

## Conflict of interest

The authors declare no competing interests.

## Author contributions

WJK and SHR: conceptualization. WJK, SHR, ZW, and HN: data curation. WJK, SHR, ZW, YW, and HN: formal analysis. WJK: funding acquisition. WJK: investigation. WJK, SHR, ZW, and HN: methodology. WJK: project administration. WJK and SHR: resources. WJK, DHL, and HN: supervision. WJK, SHR, ZW, TZ, YW, and HN: validation. WJK, SHR, ZW, YW, and HN: visualization. WJK, SHR, ZW, and YW: writing—original draft. WJK, SHR, ZW, YW, and HN: writing—review & editing.

## Declaration of generative AI and AI‐assisted technologies in the writing process

During the creation of this work, the author(s) utilized DeepSeek AI (https://chat.deepseek.com/) to rephrase English sentences and verify English grammar, as none of the authors of this paper are native English speakers. After using this tool/service, the author(s) reviewed and edited the content as needed and take(s) full responsibility for the content of the publication.

## Supporting information


**Fig. S1.** Differential regulatory element enrichment in the NPR genes, and analysis of tissue specificity for NP and NPR genes in *Drosophila melanogaster*.
**Fig. S2.** Spatial expression profile of NP and NPR genes in *D. melanogaster*.
**Fig. S3.** Transcription factors (TFs) that specifically regulate NP and NPR genes in each *Drosophila* tissue.
**Fig. S4.** Tissue‐specific TFs that regulate NP and NPR genes.
**Fig. S5.** Tissue‐specific TFs that regulate NPs and NPRs.
**Fig. S6.** Tissue‐specific TFs that regulate NPs and NPRs.
**Fig. S7.** Regulatory architecture and dynamics of TF–NP and TF–NPR networks across various cell types and developmental times of *Drosophila melanogaster*.
**Fig. S8.** Comparative analysis of TF networks in *ilp*/*InR*‐expressing cells and NP‐NPR pairs across the Head and Body regions.
**Fig. S9.** Comparative analysis of TF networks in various cells expressing different sets of NP‐NPR pairs across head and body regions of *D. melanogaster*.
**Fig. S10.** Comparative analysis of TF networks in various cells expressing different sets of NP‐NPR pairs across head and body regions of *D. melanogaster* (continuation of Fig. S9).
**Fig. S11.** Comparative analysis of TF networks in various cells expressing different sets of NP‐NPR pairs across head and body regions of *D. melanogaster* (continuation of Fig. S10).
**Fig. S12.** Relative expression changes of NP/NPR genes during temperature shifts in *D. melanogaster*.
**Fig. S13.** Network of TFs that simultaneously regulate both NP and NPR genes in the *D. melanogaster* head.
**Fig. S14.** Network of TFs that simultaneously regulate both NP and NPR genes in the *D. melanogaster* body.

**Table S1.** Annotation file of the TFs that simultaneously regulate both NP and NPR genes.

**Table S2.** Regulon information of TFs that regulate the expression of NP and NPR genes in common.

## Data Availability

Strains and plasmids are available upon request. The authors affirm that all the data essential for confirming the conclusions of the article are present within the article, figures, tables, and supplementary data. Code for data cleaning and analysis is provided as part of the replication package. Scripts specifically related to TF network analysis can be accessed at https://github.com/hcls-kimlab/NPR_Project. The repository is officially uploaded to FEBS Open Bio.
