# Supplementary material for: Transcriptional regulation of neuropeptide receptors underlies context‐dependent adaptation in Drosophila melanogaster
Source: FEBS Open Bio. 2025 Aug 21;16(1):90–115. doi: 10.1002/2211-5463.70107 (PMC12767780; doi:10.1002/2211-5463.70107)

Transcriptional regulation of neuropeptide receptors underlies context-dependent adaptation in *Drosophila melanogaster*

SeungHeui Ryu[1, †], Yanan Wei[2, †], Zekun Wu[2, †], Tianmu Zhang[2], DoHoon Lee[1, *], Hadi Najafi[3, *], and Woo Jae Kim[2, 4, *]

Supplementary fig. S1. Differential regulatory element enrichment in the NPR genes, and analysis of tissue specificity for NP and NPR genes in *Drosophila melanogaster*.

A) Significant NPR-biased enrichment of regulatory elements, represented as the number of ReMap peaks, in comparison with their ligands (NPs) whether analyzed in paired or unpaired fashions. As control of this analysis, another class of receptor proteins, called adhesion GPCRs (aGPCRs), was also investigated in parallel. No significant enrichment of such regulatory elements was observed for aGPCRs as compared to the genomic regions of NP genes.

B) Non-significant differences between the diversity of tissues expressing NP versus those expressing NPRs genes. '*' and 'ns' denote respectively the significant ($P < 0.05$) and non-significant differences.

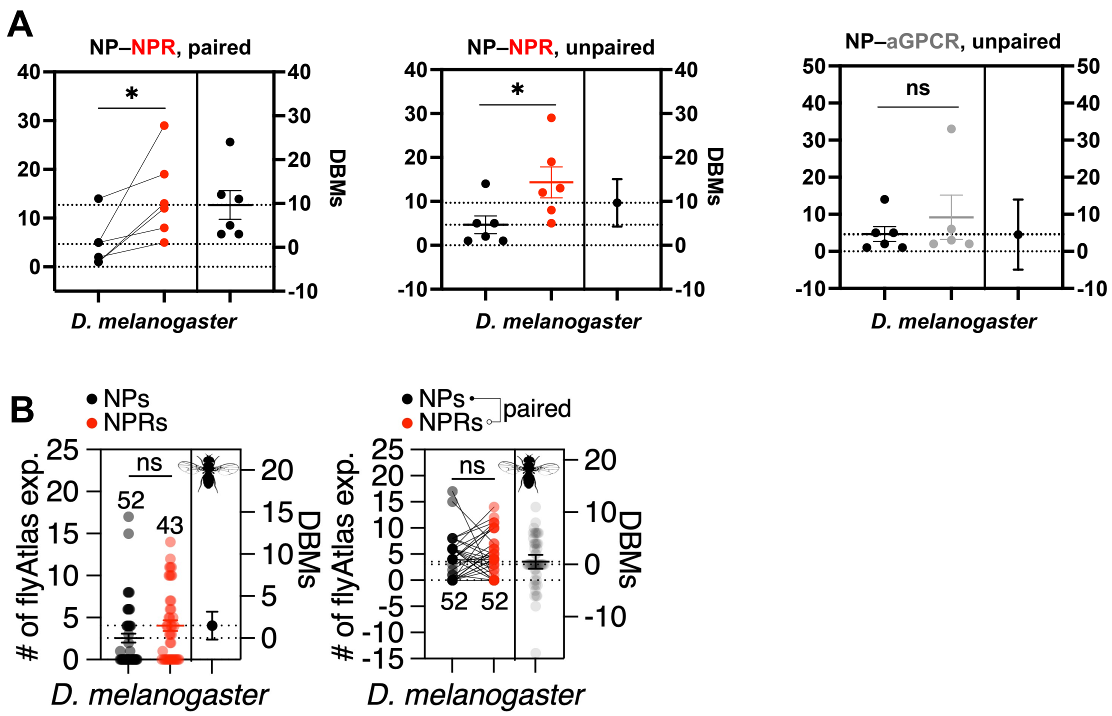

Supplementary fig. S2. Spatial expression profile of NP and NPR genes in *D. melanogaster*.

A–Q) Scatter Plot of 'total gene expression' (TPM) and 'total cell count' (TCC) by tissues in *D. melanogaster*, including Head (**A**), Body (**B**), Antenna (**C**), Body wall (**D**), Fat body (**E**), Gut (**F**), Haltere (**G**), Heart (**H**), Leg (**I**), Male reproductive glands (**J**), Malpighian tubule (**K**), Oenocyte (**L**), Ovary (**M**), Proboscis (**N**), Testis (**O**), Trachea (**P**), Wing (**Q**). Dots representing the NP or NPR genes are color-coded by cell count (C.C) threshold. The log scale on the y-axis indicates the transformation applied to the TCC values for visualization purposes.

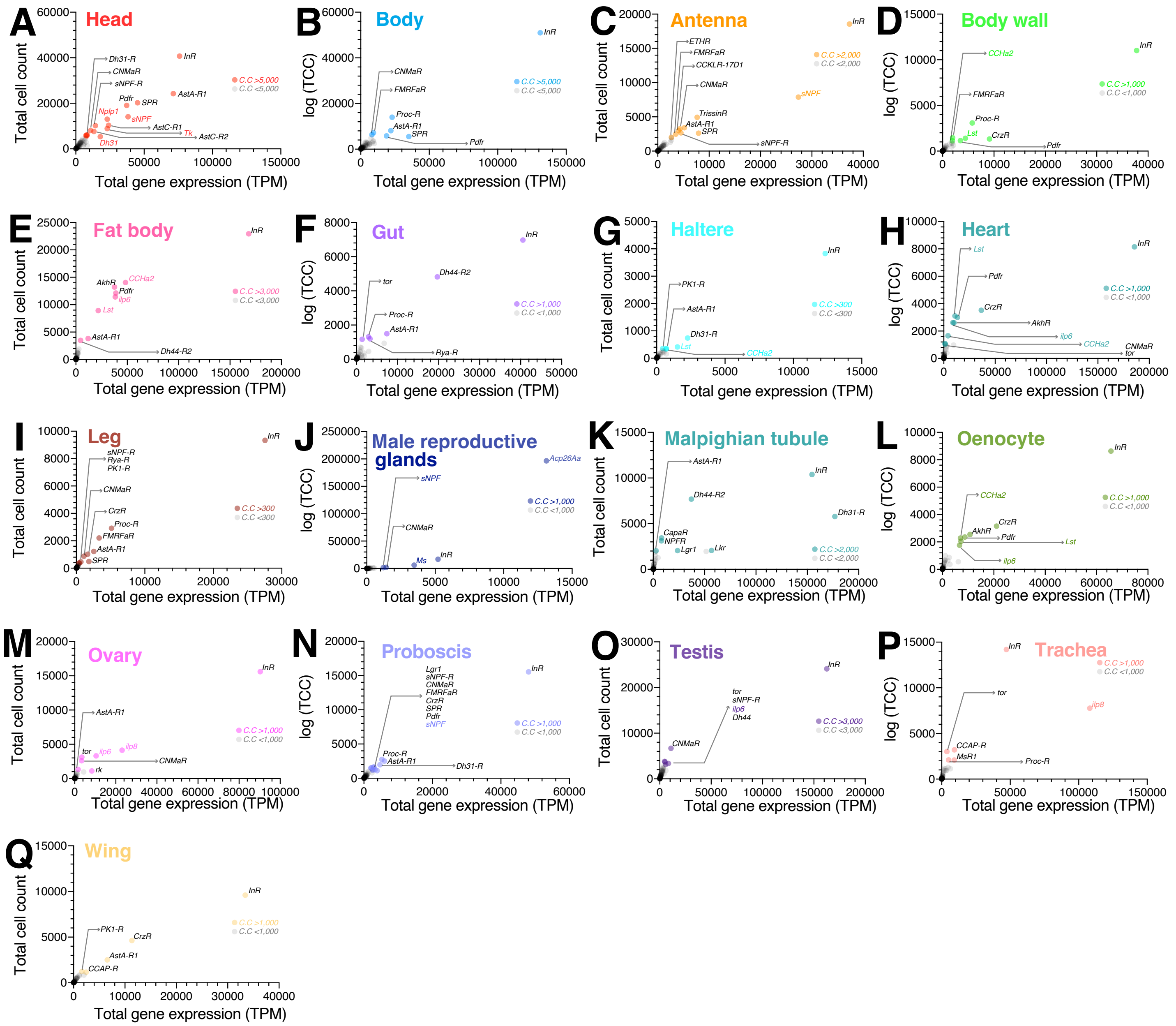

Supplementary fig. S3. Transcription factors (TFs) that specifically regulate NP and NPR genes in each *Drosophila* tissue.

A–C) Tissue-independent network of TFs and their related NPs (gray zone) and NPRs (pink zone) across 17 annotated tissues in *D. melanogaster*. The brown connecting lines (edges) denote association between the NP or NPR genes with TFs. The regulatory association of TFs with NP and NPR genes are indicated by black and red nodes, respectively. Within each network of TF-NP and TF-NPR, the NP or NPR genes that are regulated by TFs are denoted by gray and brown nodes, respectively. The 'median' and 'mean' values were calculated from the total number of individual expressions of representative NP and NPR genes. The data was constructed by using the 'MotifRegulon' information from each of the 17 tissue cells to identify and connect NPs with their TFs as well as connecting NPRs with their TFs within the same cell.

D–F) Tissue independent network of TFs with NP and NPR genes by excluding the common TFs, expressed both NP- and NPR-expressing cells. Details of the analysis and the networks are the same as described above (A–C).

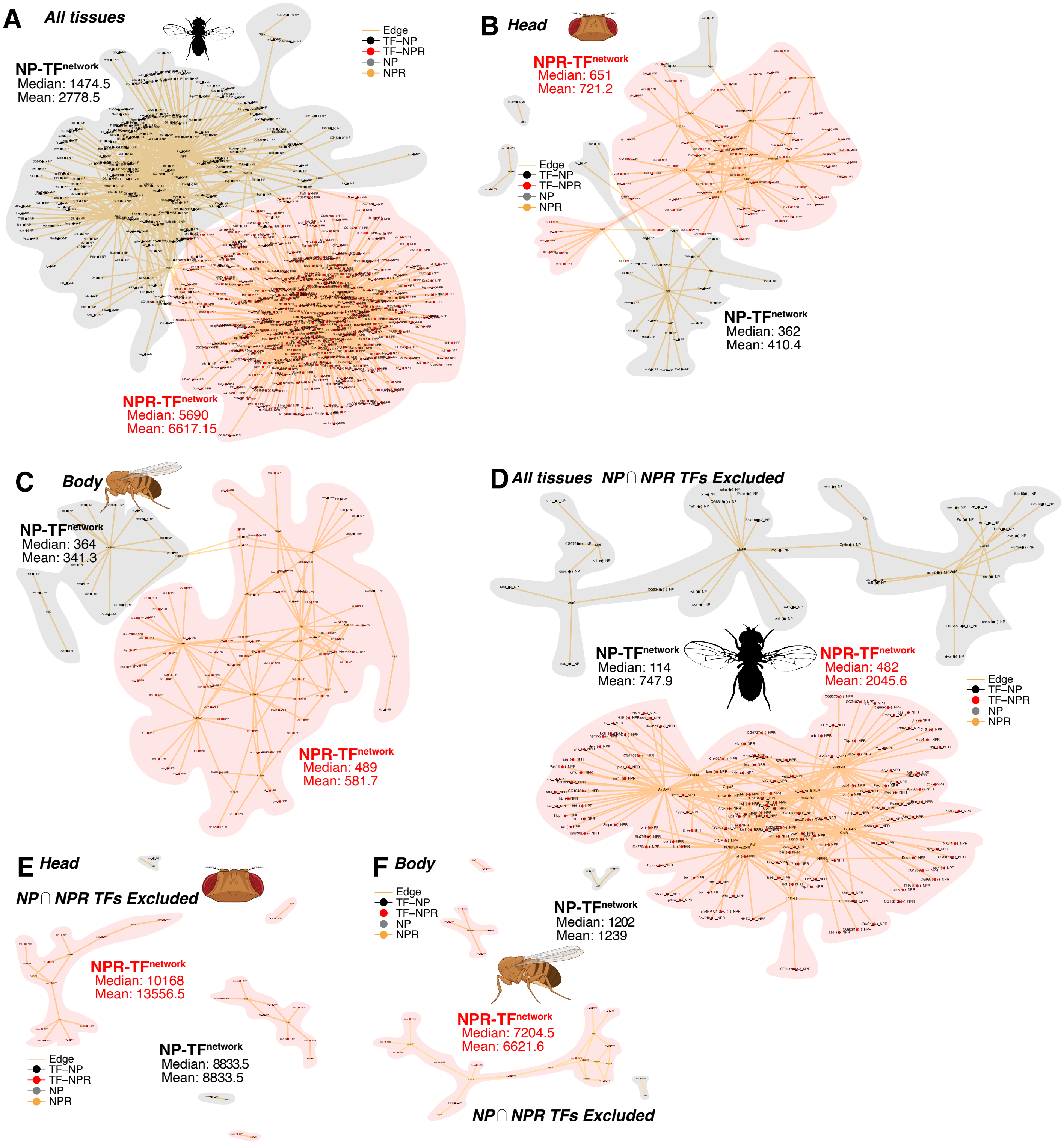
A
All tissues
NP-TFnetwork
Median: 1474.5
Mean: 2778.5
NPR-TFnetwork
Median: 5690
Mean: 6617.15
Edge
TF–NP
TF–NPR
NP
NPR
B
Head
NPR-TFnetwork
Median: 651
Mean: 721.2
NP-TFnetwork
Median: 362
Mean: 410.4
C
Body
NP-TFnetwork
Median: 364
Mean: 341.3
NPR-TFnetwork
Median: 489
Mean: 581.7
D
All tissues NP∩NPR TFs Excluded
NP-TFnetwork
Median: 114
Mean: 747.9
NPR-TFnetwork
Median: 482
Mean: 2045.6
E
Head
NP∩NPR TFs Excluded
NPR-TFnetwork
Median: 10168
Mean: 13556.5
NP-TFnetwork
Median: 8833.5
Mean: 8833.5
F
Body
NP-TFnetwork
Median: 1202
Mean: 1239
NPR-TFnetwork
Median: 7204.5
Mean: 6621.6
NP∩NPR TFs Excluded

Supplementary fig. S4. Tissue-specific TFs that regulate NP and NPR genes.

A) Network of TFs that regulate NPs and NPRs in the fat body (the details are the same as described for the supplementary fig. S3A–C).

B) Number of TFs that regulate NPs and NPRs in the fat body.

C) Number of NPs and NPRs regulated by TFs in the fat body**.**

D) Network of TFs that regulate NPs and NPRs in the Gut (the details are the same as described for the supplementary fig. S3A–C).

E) Number of TFs that regulate NPs and NPRs in the Gut.

F) Number of NPs and NPRs regulated by TFs in the Gut.

G) Network of TFs that regulate NPs and NPRs in the Heart (the details are the same as described for the supplementary fig. S3A–C).

H) Number of TFs that regulate NPs and NPRs in the Heart.

I) Number of NPs and NPRs regulated by TFs in the Heart.

J) Network of TFs that regulate NPs and NPRs in the Malpighian tubules (the details are the same as described for the supplementary fig. S3A–C).

K) Number of TFs that regulate NPs and NPRs in the Malpighian tubules.

L) Number of NPs and NPRs regulated by TFs Malpighian tubules.

M) Network of TFs that regulate NPs and NPRs in the Oenocyte (the details are the same as described for the supplementary fig. S3A–C).

N) Number of TFs that regulate NPs and NPRs in the Oenocyte.

O) Number of NPs and NPRs regulated by TFs in the Oenocyte.

P) Network of TFs that regulate NPs and NPRs in the Trachaea (the details are the same as described for the supplementary fig. S3A–C).

Q) Number of TFs that regulate NPs and NPRs in the Trachaea.

R) Number of NPs and NPRs regulated by TFs in the Trachaea.

For the graphs, the unpaired t-test was used to evaluate the statistical significance of the data (* = $P < 0.05$, ** = $P < 0.01$, *** = $P < 0.001$, **** = $P < 0.0001$). Non-significant changes are indicated by 'ns'.

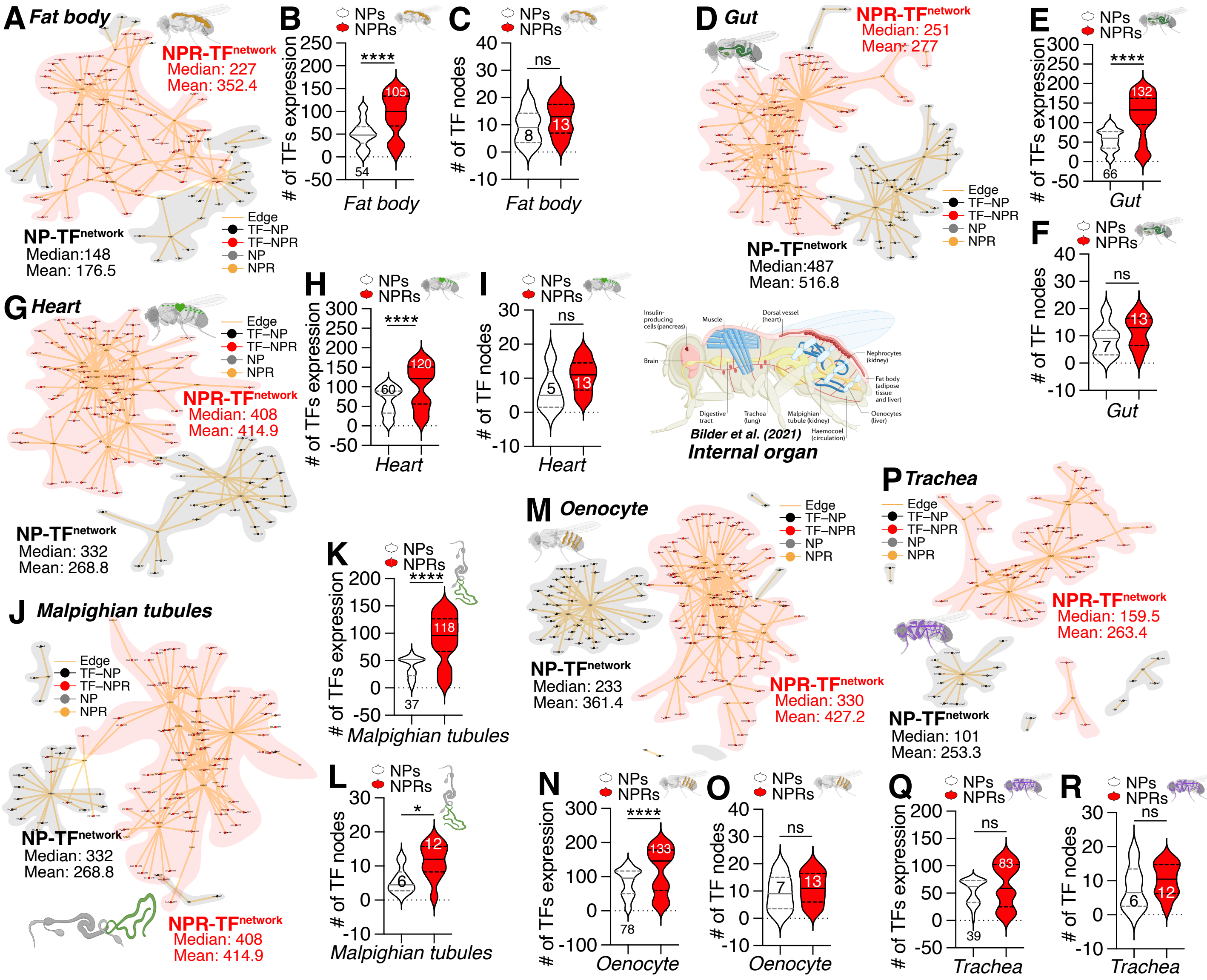

Supplementary fig. S5. Tissue-specific TFs that regulate NPs and NPRs.

A) Network of TFs that regulate NPs and NPRs in the Antenna (the details are the same as described for the supplementary fig. S3A–C).

B) Number of TFs that regulate NPs and NPRs in the Antenna.

C) Number of NPs and NPRs regulated by TFs in the Antenna.

D) Network of TFs that regulate NPs and NPRs in the Body wall (the details are the same as described for the supplementary fig. S3A–C).

E) Number of TFs that regulate NPs and NPRs in the Body wall.

F) Number of NPs and NPRs regulated by TFs in the Body wall.

G) Network of TFs that regulate NPs and NPRs in the Haltere (the details are the same as described for the supplementary fig. S3A–C).

H) Number of TFs that regulate NPs and NPRs in the Haltere.

I) Number of NPs and NPRs regulated by TFs in the Haltere.

J) Network of TFs that regulate NPs and NPRs in the Leg (the details are the same as described for the supplementary fig. S3A–C).

K) Number of TFs that regulate NPs and NPRs in the Leg.

L) Number of NPs and NPRs regulated by TFs Leg.

M) Network of TFs that regulate NPs and NPRs in the Proboscis (the details are the same as described for the supplementary fig. S3A–C).

N) Number of TFs that regulate NPs and NPRs in the Proboscis.

O) Number of NPs and NPRs regulated by TFs in the Proboscis.

P) Network of TFs that regulate NPs and NPRs in the Wing (the details are the same as described for the supplementary fig. S3A–C).

Q) Number of TFs that regulate NPs and NPRs in the Wing.

R) Number of NPs and NPRs regulated by TFs in the Wing.

For the graphs, the unpaired t-test was used to evaluate the statistical significance of the data (* = $P < 0.05$, ** = $P < 0.01$, *** = $P < 0.001$, **** = $P < 0.0001$). Non-significant changes are indicated by ‘ns’.

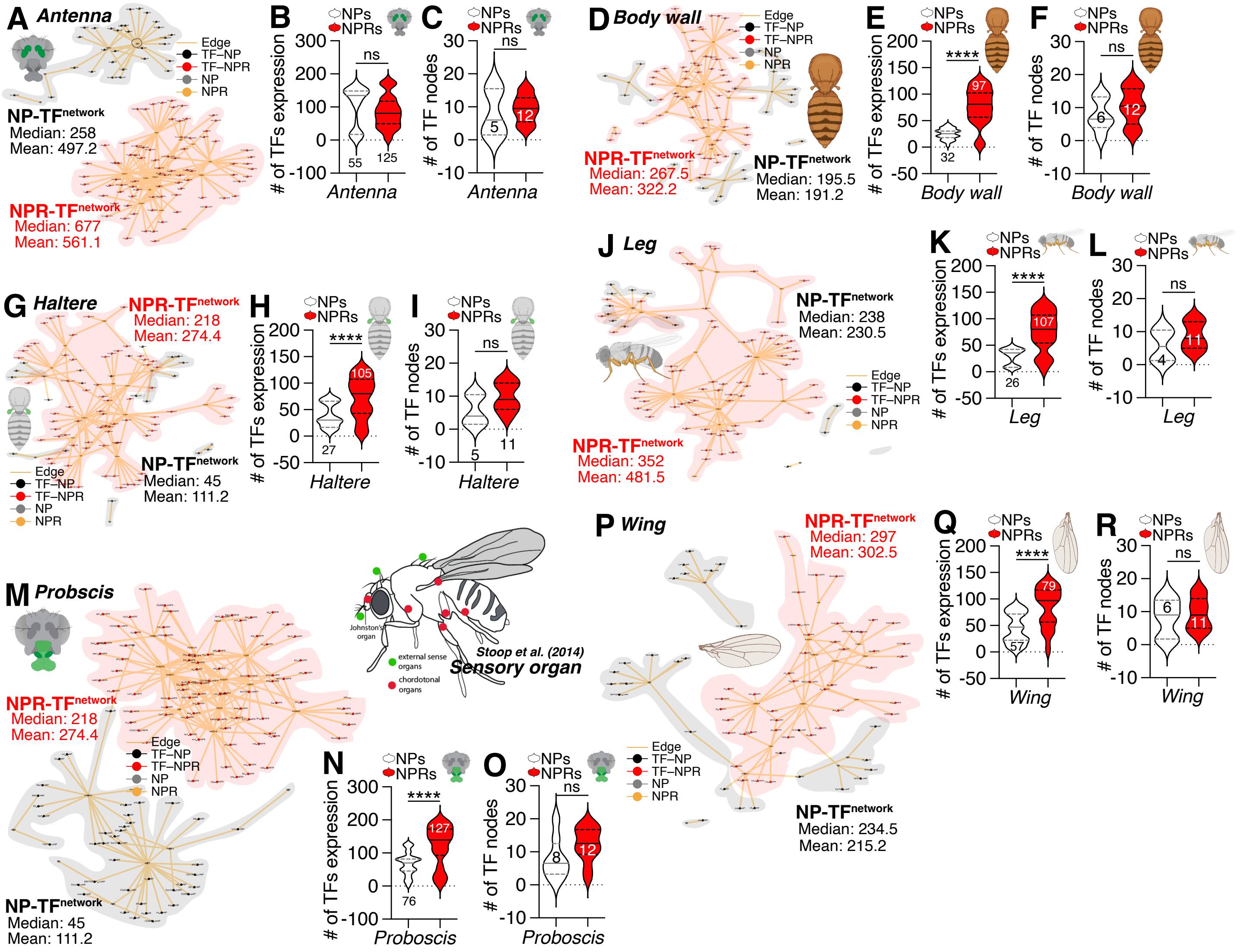

Supplementary fig. S6. Tissue-specific TFs that regulate NPs and NPRs.

A) Network of TFs that regulate NPs and NPRs in the Male reproductive glands (the details are the same as described for the supplementary fig. S3A–C).

B) Number of TFs that regulate NPs and NPRs in the Male reproductive glands.

C) Number of NPs and NPRs regulated by TFs in the Male reproductive glands.

D) Network of TFs that regulate NPs and NPRs in the Ovary (the details are the same as described for the supplementary fig. S3A–C).

E) Number of TFs that regulate NPs and NPRs in the Ovary.

F) Number of NPs and NPRs regulated by TFs in the Ovary.

G) Network of TFs that regulate NPs and NPRs in the Testis (the details are the same as described for the supplementary fig. S3A–C).

H) Number of TFs that regulate NPs and NPRs in the Testis.

I) Number of NPs and NPRs regulated by TFs in the Testis.

For the graphs, the unpaired t-test was used to evaluate the statistical significance of the data (* = $P < 0.05$, ** = $P < 0.01$, *** = $P < 0.001$, **** = $P < 0.0001$). Non-significant changes are indicated by 'ns'.

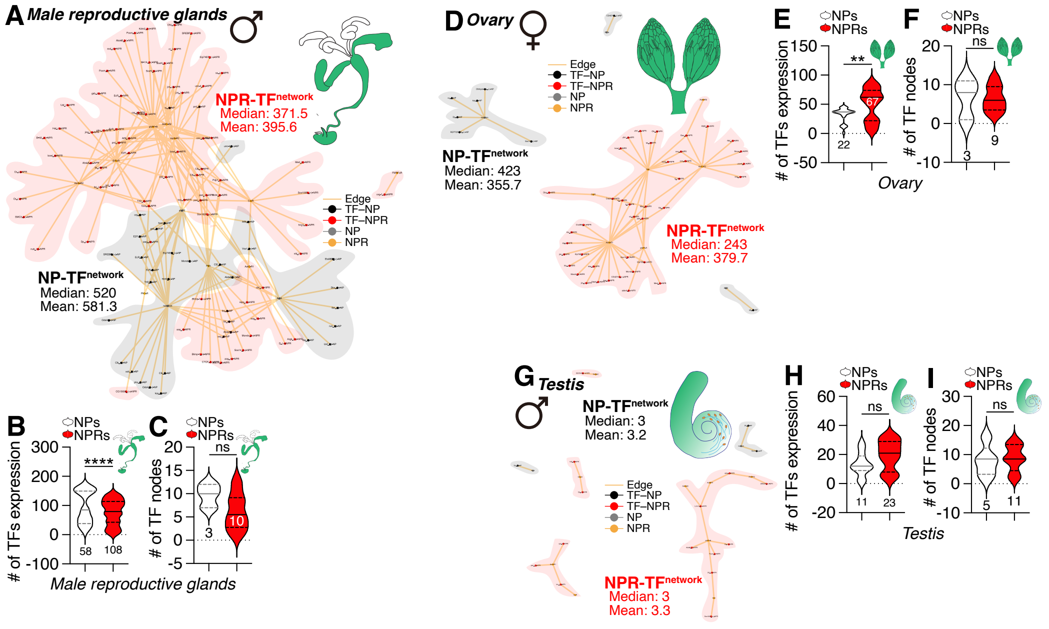

Supplementary fig. S7. Regulatory architecture and dynamics of TF–NP and TF–NPR networks across various cell types and developmental times of *Drosophila melanogaster*.

A–L) Cell type-specific regulatory dynamics of TF–NP and TF–NPR interactions. Regulatory networks in different neuronal subtypes: antennal lobe projection neuron (**A** & **G**), dopaminergic PAM neuron (**B** & **H**), gustatory receptor neuron (**C** & **I**), Kenyon cell (**D** & **J**), octopaminergic/tyraminergic neuron (**E** & **K**), and olfactory receptor neuron (**F** & **L**). Left graphs (**A–F**) represent the Dual Top 50 Ranking (TF–NP vs. TF–NPR) – Comparison of the top 50 most influential TF–NP pairs (gray bars) and TF–NPR pairs (red bars) at each age (5, 30, 50, 70 days post-eclosion). Right graphs (**G–L**) represent the Combined Top 100 TF–NP/NPR Ranking – Pooled analysis of the 100 highest-ranked TF–NP or TF–NPR pairs across ages. Asterisks represent significant differences (*p < 0.05, **p < 0.01, ***p < 0.001, ****p < 0.0001), as revealed by the unpaired Student's t test. 'ns' indicates non-significant differences.

M) Number of cells (cell count) with expression of NP and NPR genes during aging of *D. melanogaster*.

N) Network of TFs that regulate NP and NPR genes in all tissues on Day 1 (the details are the same as described for the supplementary fig. S3A–C).

O) Number of TFs that regulate NPs and NPRs in all tissues on Day 1.

P) Number of NPs and NPRs regulated by TFs in all tissues on Day 1.

Q) Network of TFs that regulate NPs and NPRs in all tissues on Day 3 (the details are the same as described for the supplementary fig. S3A–C).

R) Number of TFs that regulate NPs and NPRs in all tissues on Day 3.

S) Number of NPs and NPRs regulated by TFs in all tissues on Day 3.

T) Number of TFs remaining after removing those expressed in Day 3 from Day 5 (denoted by day 5^{-3}), and those expressed on Day 3 after subtracting TFs expressed in Day 1 from them (denoted by day 3^{-1}).

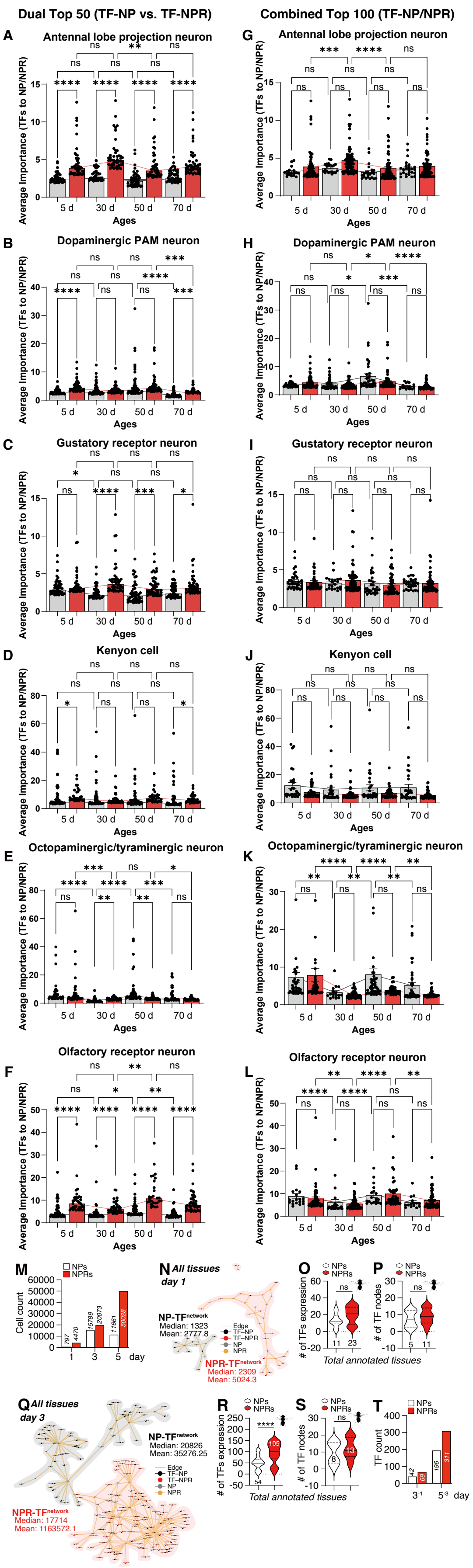

Supplementary fig. S8. Comparative analysis of TF networks in *ilp*/*InR*-expressing cells and NP-NPR pairs across the Head and Body regions.

A–E) Characteristics of the TF networks in *ilp*- and *InR*-expressing cells, including quantification (number) of TF-regulated cell counts in *ilp*-positive (white bars) and *InR*-positive (red bars) cells across total, head, and body parts of *D. melanogaster* (**A**). Network maps of TFs in *ilp*- and *InR*-expressing cells in the head (**B**) and body (**D**) tissues, and violin plots (**C** and **E**) show the pattern of TF distribution in the *ilp*- and *InR*-expressing cells among head (**B–C**) and body (**D–E**) parts.

F-U) Differences in the number of regulatory TFs which are expressed in the *Drosophila* head and body regions, across various sets of NP-NPR pairs.

For the graphs, the unpaired t-test was used to evaluate the statistical significance of the data (* = $P < 0.05$, ** = $P < 0.01$, *** = $P < 0.001$, **** = $P < 0.0001$). Non-significant changes are indicated by 'ns'.

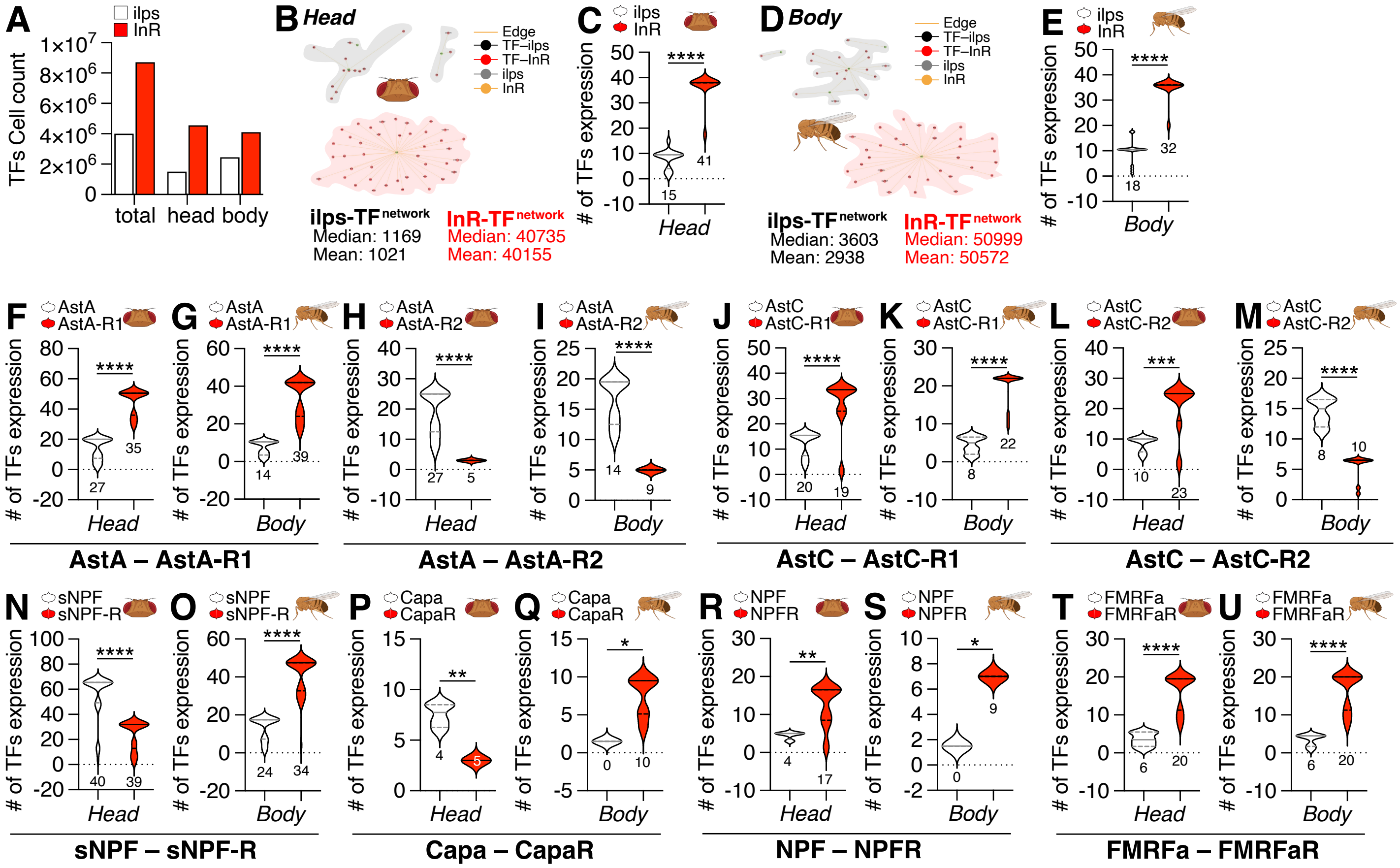

Supplementary fig. S9. Comparative analysis of TF networks in various cells expressing different sets of NP-NPR pairs across head and body regions of *D. melanogaster*.

A–B) *Acp26aAa–SPR*, **C–D**) *Akh–AkhR*, **E–F**) *Burs–rk*, **G–H**) *CCAP–CCAP-R*, **I–J**) *CNMa–CNMaR*, K-L) *ETH–ETHR*, **M–N**) *CCHa1–CCHa1-R*, **O–P**) *CCHa2–CCHa2-R*, **Q–R**) *Dh31–hec*, **S–T**) *Dh44–Dh44-R1*, **U–V**) *Dsk–CCKLR-17D3*, and **W–X**) *Gbp5–Lgr1*.

For the graphs, the unpaired t-test was used to evaluate the statistical significance of the data (* = $P < 0.05$, ** = $P < 0.01$, *** = $P < 0.001$, **** = $P < 0.0001$). Non-significant changes are indicated by 'ns'.

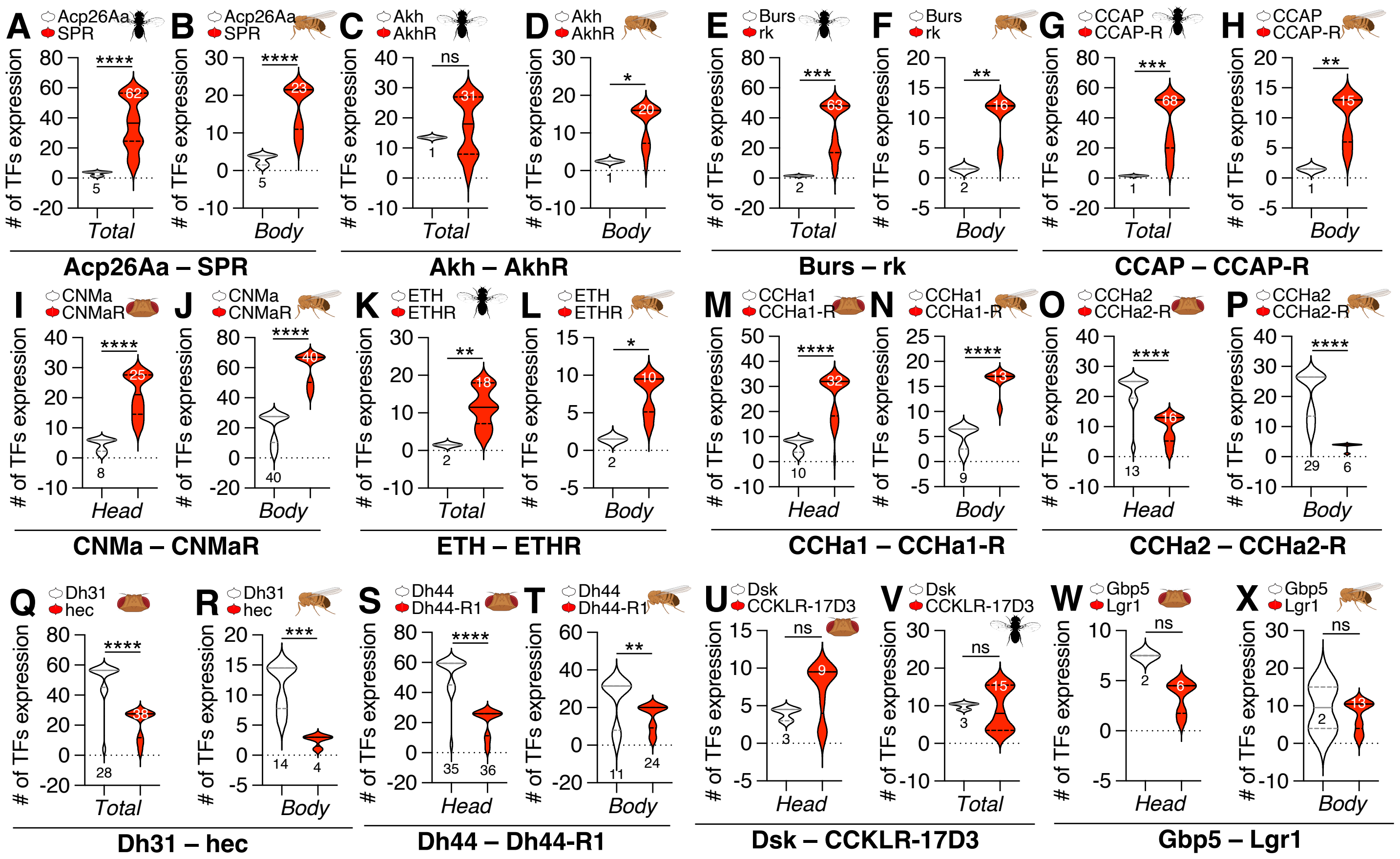

Supplementary fig. S10. Comparative analysis of TF networks in various cells expressing different sets of NP-NPR pairs across head and body regions of *D. melanogaster* (continuation of fig. S9).

A–B) *Tk–TkR99D*, **C–D**) *Tk–TkR86C*, **E–F**) *natalisin–TkR86C*, **G–H**) *Hug–PK2-R1*, **I–J**) *Hug–PK2-R2*, **K–L**) *Ms–MsR1*, **M––N**) *Ms–MsR2*, **O–P**) *ilp7–Lgr4*, and **Q–R**) *ilp8–Lgr3*.

For the graphs, the unpaired t-test was used to evaluate the statistical significance of the data (* = $P < 0.05$, ** = $P < 0.01$, *** = $P < 0.001$, **** = $P < 0.0001$). Non-significant changes are indicated by 'ns'.

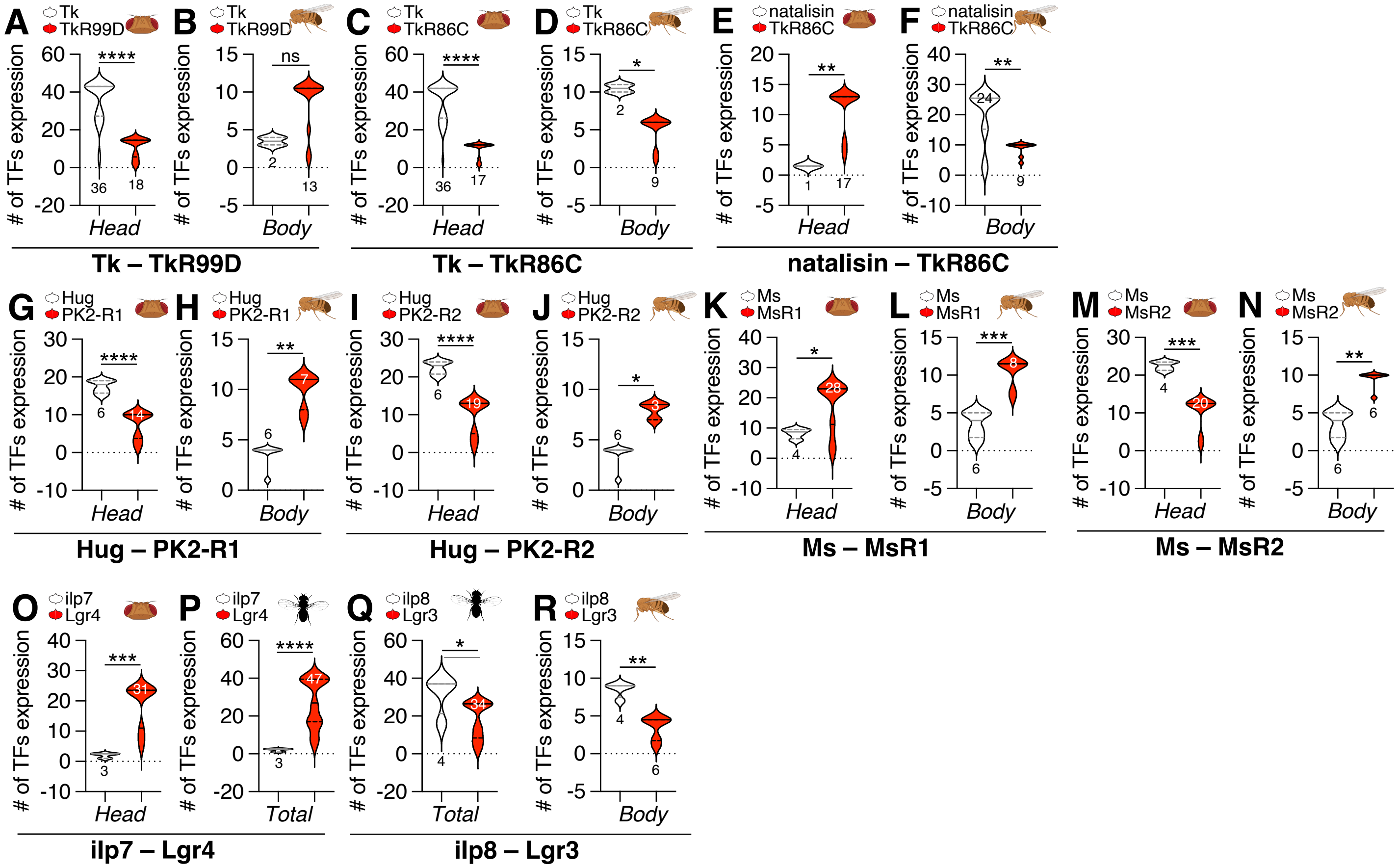

Supplementary fig. S11. Comparative analysis of TF networks in various cells expressing different sets of NP-NPR pairs across head and body regions of *D. melanogaster* (continuation of fig. S10).

A–B) *Proc–ProcR*, **C–D)** *Nplp1–Gyc76C*, **E–F)** *Trissin–TrissinR*, **G–H)** *Pdf–Pdfr*, **I–J)** *SIFa–SIFaR*, **K–L)** *Crz–CrzR*, **M–N)** *Lk–Lkr*, and **O–P)** *Ptth–Tor*.

For the graphs, the unpaired t-test was used to evaluate the statistical significance of the data (* = $P < 0.05$, ** = $P < 0.01$, *** = $P < 0.001$, **** = $P < 0.0001$). Non-significant changes are indicated by 'ns'.

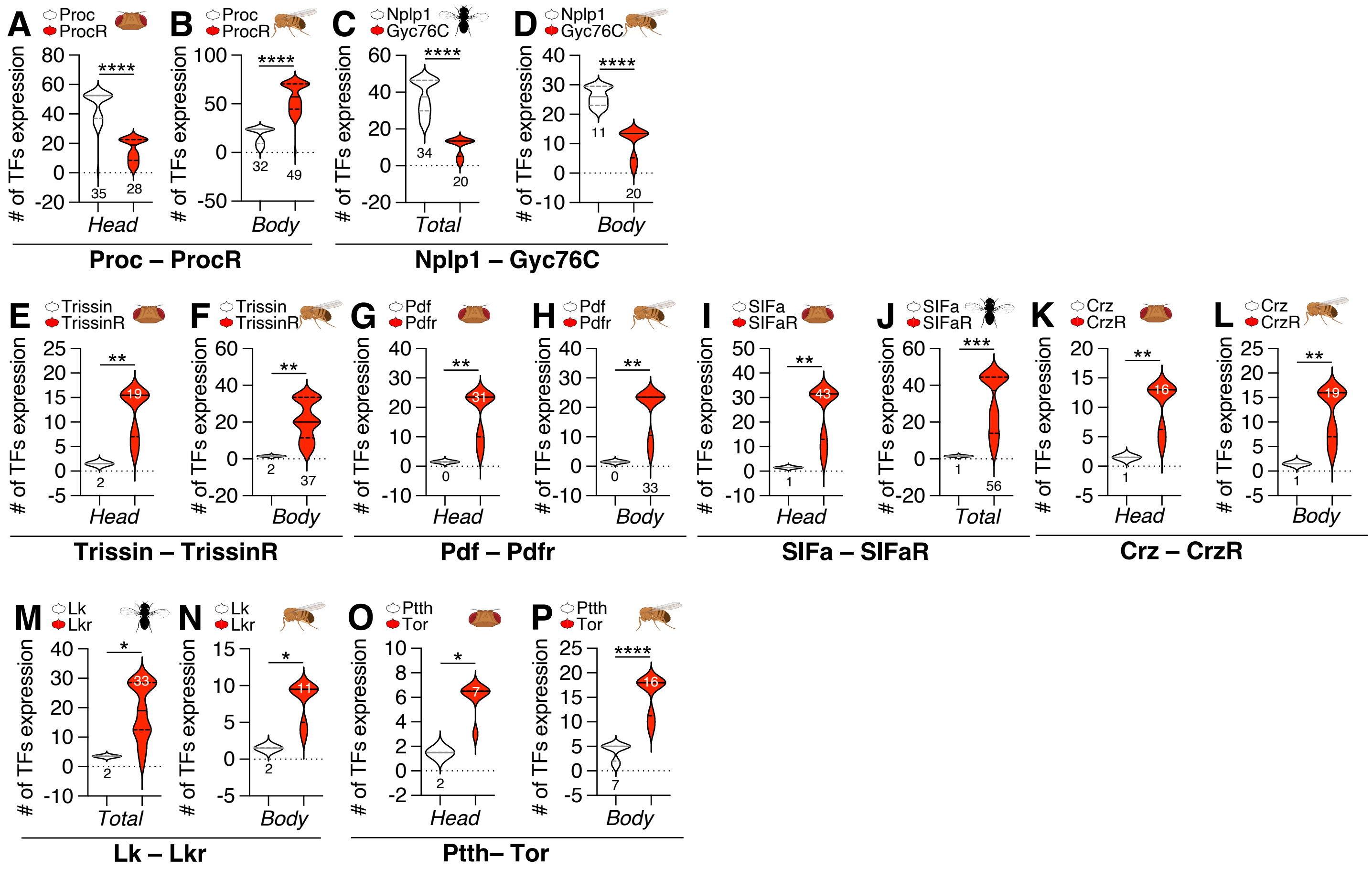

Supplementary fig. S12. Relative expression changes of NP/NPR genes during temperature shifts in *D. melanogaster.*

A–D) Expression change of candidate NP/NPR pairs (*AstA, Crz, SIFa*, *sNPF, AstA-R1*, *AstA-R2, Crz-R, SIFa-R* and *sNPF-R*) in adult Canton-S female flies rearing in different temperature conditions. For this quantification, qRT-PCR was performed in three independent experiments and *GAPDH* gene was used as internal control for gene expression normalization. Expression level changes of NP/NPR genes were studied in both *Drosophila* body and head tissues separately, as indicated on top of each graph.

E–H) Expression change of candidate NP/NPR pairs (*AstA, Crz, SIFa*, *sNPF, AstA-R1*, *AstA-R2, Crz-R, SIFa-R* and *sNPF-R*) in adult Canton-S male flies rearing in different temperature conditions. For this quantification, qRT-PCR was performed in three independent experiments and *GAPDH* gene was used as internal control for gene expression normalization. Expression level changes of NP/NPR genes were studied in both *Drosophila* body and head tissues separately, as indicated on top of each graph.

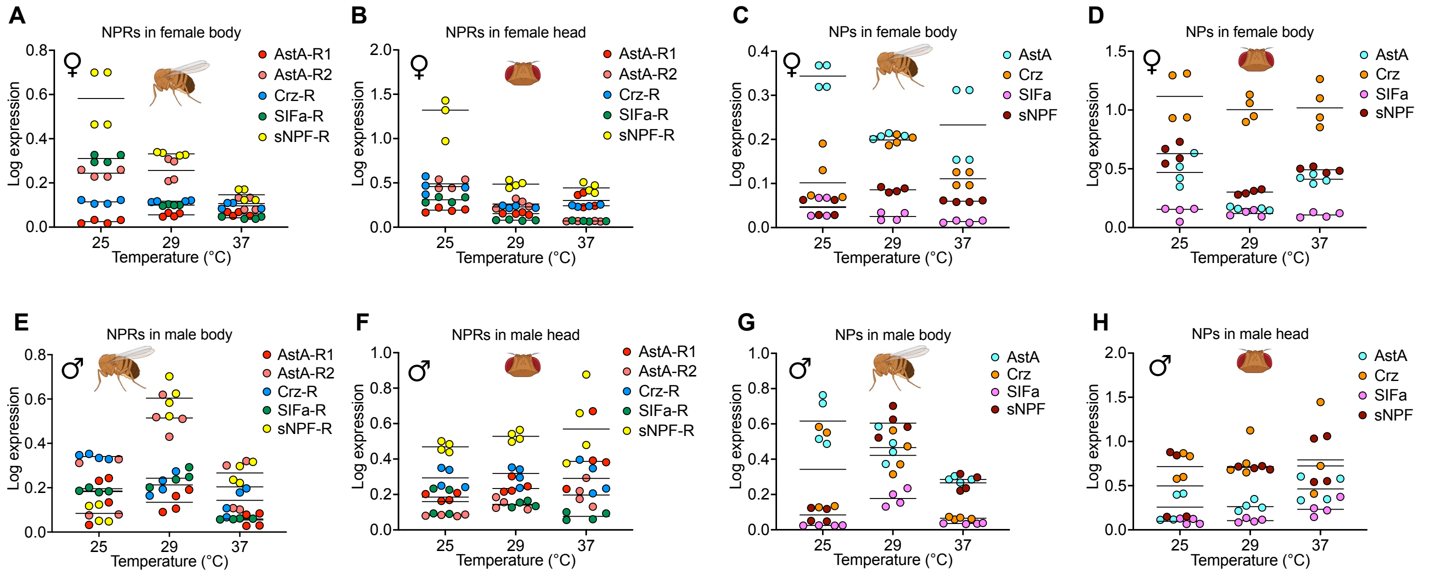

Supplementary fig. S13. Network of TFs that simultaneously regulate both NP and NPR genes in the *D. melanogaster* head.

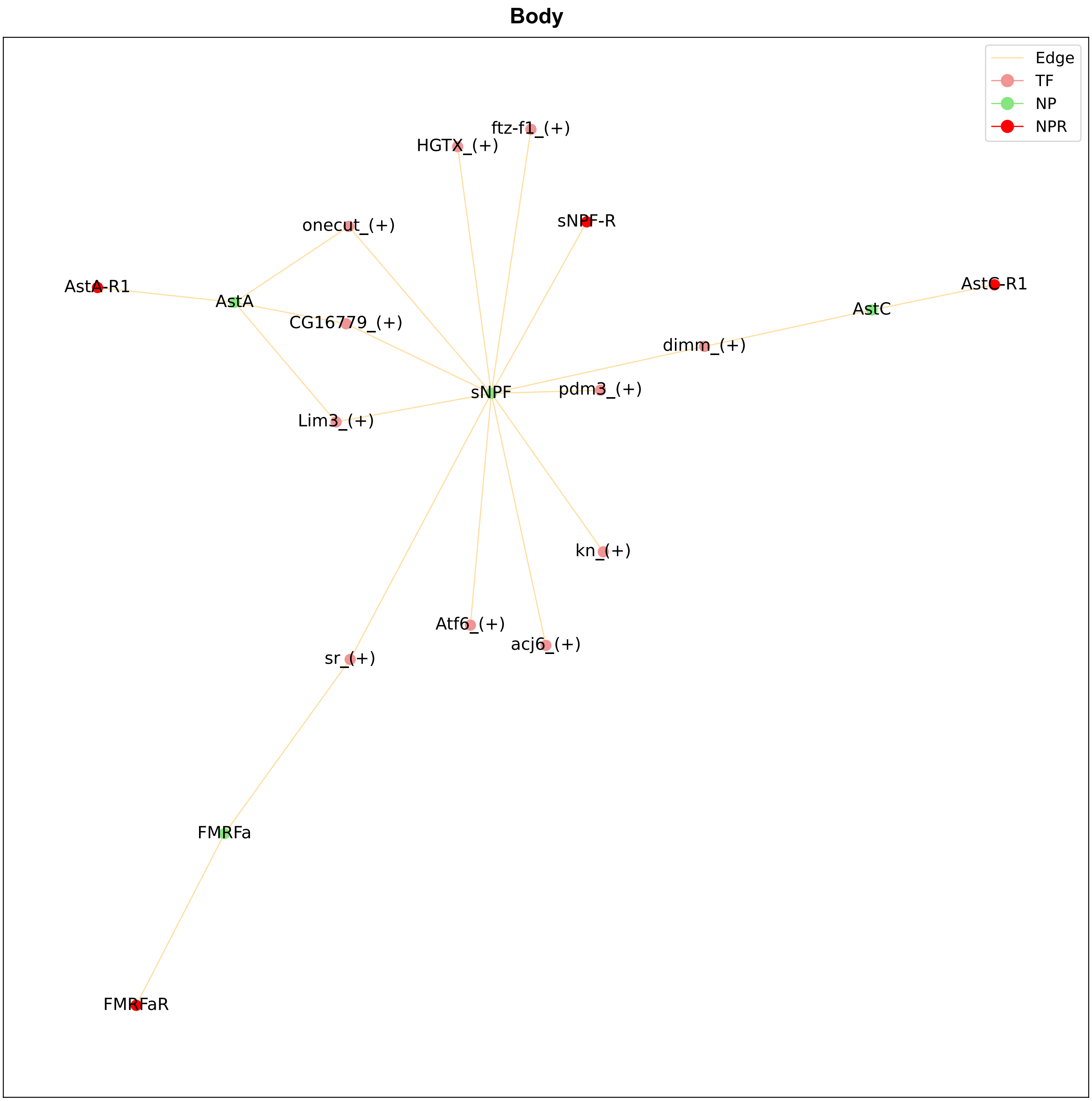

Supplementary fig. S14. Network of TFs that simultaneously regulate both NP and NPR genes in the *D. melanogaster* body.

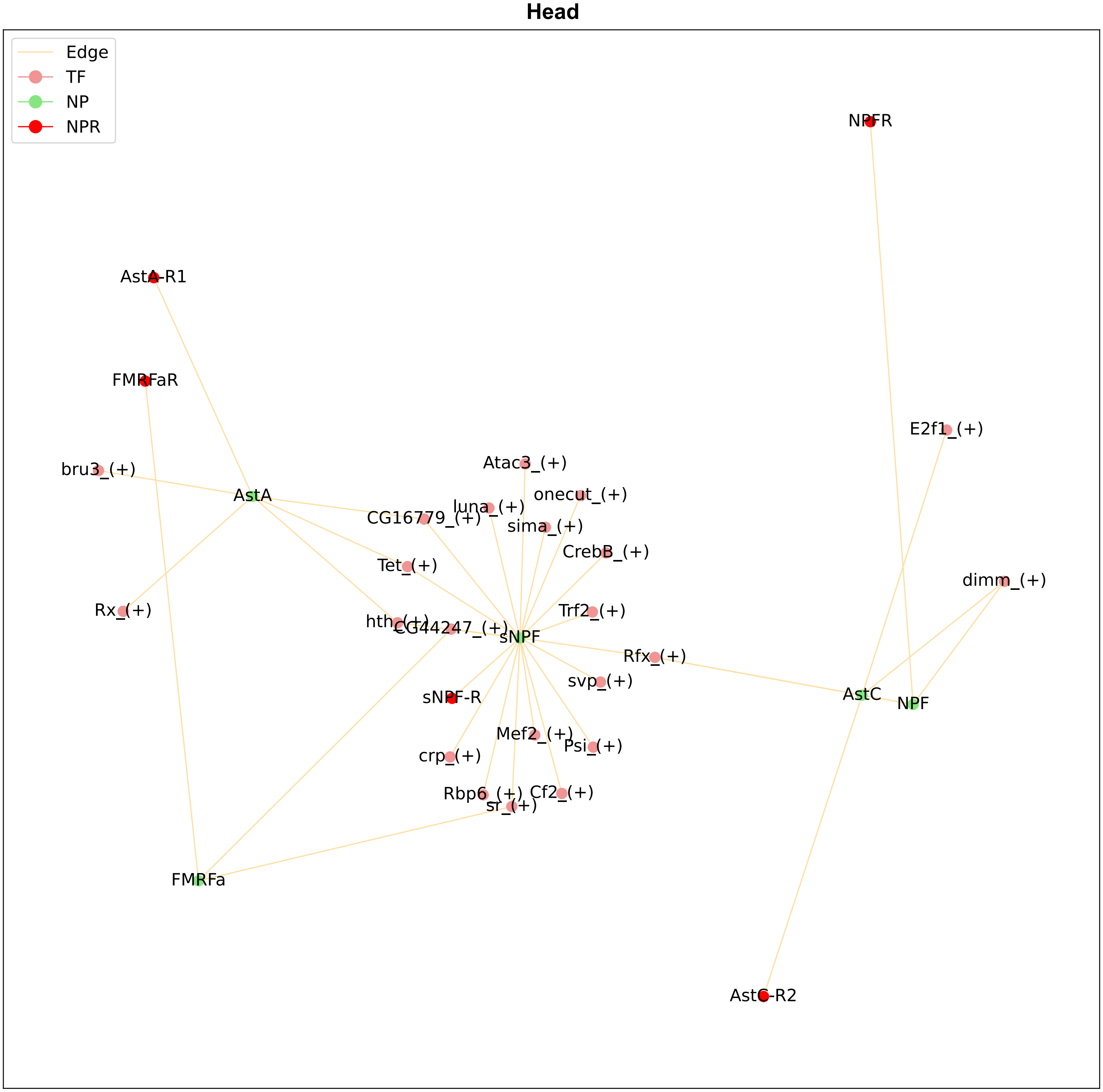

Supplement: Supplementary file 1 — Fig. S1. Differential regulatory element enrichment in the NPR genes, and analysis of tissue specificity for NP and NPR genes in Drosophila melanogaster. Fig. S2. Spatial expression profile of NP and NPR genes in D. melanogaster. Fig. S3. Transcription factors (TFs) that specifically regulate NP and NPR genes in each Drosophila tissue. Fig. S4. Tissue‐specific TFs that regulate NP and NPR genes. Fig. S5. Tissue‐specific TFs that regulate NPs and NPRs. Fig. S6. Tissue‐specific TFs that regulate NPs and NPRs. Fig. S7. Regulatory architecture and dynamics of TF–NP and TF–NPR networks across various cell types and developmental times of Drosophila melanogaster. Fig. S8. Comparative analysis of TF networks in ilp/InR‐expressing cells and NP‐NPR pairs across the Head and Body regions. Fig. S9. Comparative analysis of TF networks in various cells expressing different sets of NP‐NPR pairs across head and body regions of D. melanogaster. Fig. S10. Comparative analysis of TF networks in various cells expressing different sets of NP‐NPR pairs across head and body regions of D. melanogaster (continuation of Fig. S9). Fig. S11. Comparative analysis of TF networks in various cells expressing different sets of NP‐NPR pairs across head and body regions of D. melanogaster (continuation of Fig. S10). Fig. S12. Relative expression changes of NP/NPR genes during temperature shifts in D. melanogaster. Fig. S13. Network of TFs that simultaneously regulate both NP and NPR genes in the D. melanogaster head. Fig. S14. Network of TFs that simultaneously regulate both NP and NPR genes in the D. melanogaster body. [file FEB4-16-90-s002.pdf]
